# Circular Carbon Nanotube Production for Advanced Structural Fiber Applications

**DOI:** 10.1021/acsami.5c16868

**Published:** 2025-11-20

**Authors:** Xiao Sun, Di Chang, Varunkumar Thippanna, Xiaoli Li, Aidin Panahi, Huidong Dai, Hongyi Wang, Jianlin Li, Yunzheng Yang, Arunachalam Ramanathan, Wentao Liang, Yiannis Levendis, Kenan Song, Marilyn Minus

**Affiliations:** † Department of Mechanical and Industrial Engineering, 1848 Northeastern University, 360 Huntington Avenue, Boston, Massachusetts 02115, United States; ‡ Mechanical Engineering, College of Engineering, 1355 University of Georgia, 302 E Campus Rd, Athens, Georgia 30602, United States; § Chemical Engineering Department, 8718 Worcester Polytechnic Institute, Worcester, Massachusetts 01609, United States; ∥ Department of Chemistry and Chemical Biology, Northeastern University, Boston, Massachusetts 02115, United States; ⊥ Kennedy College of Sciences, 14710 University of Massachusetts Lowell, 220 Pawtucket Street, Lowell, Massachusetts 01854, United States; # Kostas Advanced Nanocharacterization Facility (KANCF), Northeastern University, Burlington, Massachusetts 01803, United States; ¶ Mechanical Engineering, College of Engineering, University of Georgia (UGA), 302 E. Campus Rd., Athens, Georgia 30602, United States

**Keywords:** postconsumer plastic, carbon nanotube, chemical vapor deposition, polyacrylonitrile fibers, stainless steel catalyst

## Abstract

Plastic waste accumulation remains a global challenge, with only a small fraction currently being recycled. Upcycling postconsumer plastics into value-added materials offers a sustainable solution for waste reduction and advanced manufacturing. In this study, we demonstrate a cost-effective method for synthesizing multiwalled carbon nanotubes (MWCNTs) from postconsumer plastics via catalytic chemical vapor deposition. The waste-derived MWCNTs were incorporated into polyacrylonitrile (PAN) through wet spinning to fabricate high-performance composite fibers. Compared to conventional PAN-based fibers that use commercially sourced CNTs, which often contain residual catalysts that weaken performance, the synthesized MWCNTs exhibit lower metal content and enhanced crystallinity (66%), reducing defects and improving composite integrity. The resulting PAN–MWCNT fibers achieved a Young’s modulus of 13.4 GPa and tensile strength of 941 MPa. This work provides a dual benefit: mitigating plastic waste while producing high-value carbon nanomaterials, offering a sustainable route to manufacture lightweight, high-performance fibers applicable in aerospace, automotive, and energy sectors.

## Graphical abstract

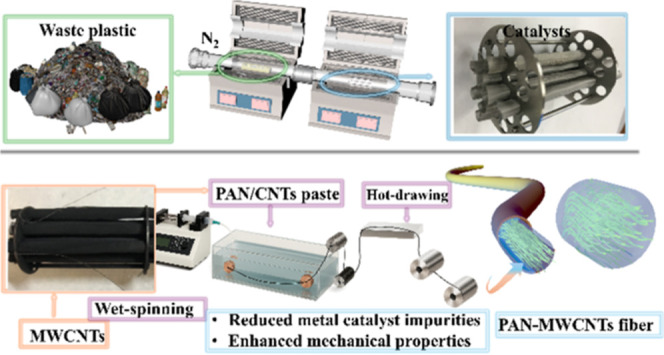

## 1 Introduction

Plastic waste has become a critical global environmental challenge due to its widespread use and low degradation rates.^1–3^ Since the mid-20th century, plastics have been integral to industries such as packaging, automotive, electronics, and healthcare, primarily because of their low cost, lightweight, and versatility.^4,5^ However, the rise of disposable plastics has led to severe ecological issues, including microplastic pollution, soil contamination, marine plastic accumulation, and greenhouse gas emissions.^6,7^ Traditional recycling methods, such as mechanical and physical recovery, offer limited efficacy due to high sorting cost and cross contaminations among solid wastes. In contrast, chemical recycling—particularly pyrolysis—presents a promising route to convert plastic waste into high-value carbon materials, including graphite and carbon nanotubes (CNTs).^8,9^ Given the high carbon and hydrogen content of plastics, this approach not only mitigates plastic waste but also creates an opportunity to upcycle waste streams into functional nanomaterials for advanced applications (e.g., in defense, construction, and energy storage).^3,10,11^

CNTs are typically synthesized via several methods, including arc discharge, laser ablation, and chemical vapor deposition (CVD).^12^ Among these, CVD is the most scalable and industrially viable due to its controllable reaction parameters, lower operating costs, and ability to tailor CNT structure, purity, and yield.^13,14^ Unlike arc discharge and laser ablation, which require high temperatures and produce significant byproducts, CVD allows for direct growth of CNTs on catalyst substrates using hydrocarbon feedstocks, making it particularly suitable for integrating waste-derived carbon sources into CNT production.^15–17^ In addition, CNTs are of particular interest for next-generation structural fibers due to their extraordinary mechanical strength, electrical conductivity, and thermal stability.^18–23^ These properties make CNTs as valuable reinforcement agents in polyacrylonitrile (PAN)-based composite fibers, primary precursors for carbon fibers (CFs) due to high carbon yield.^24^ Incorporating CNTs into PAN fibers can enhance crystallization, alignment, and interfacial load transfer, resulting in improved tensile strength, modulus, and fatigue resistance.^25,26^ Also, higher crystallinity in PAN–CNT composites can reduce heat treatment temperatures required for CF production (i.e., fewer defects), lowering energy consumption and cost without sacrificing mechanical performance.^15,27–37^ This solution offers dual benefits of enhancing CF properties while addressing economic challenges in CF industry.^38–44^

Despite this potential, challenges such as uniform CNT dispersion and residual catalyst impurities from conventional CNT sources often limit the effectiveness of CNT-reinforced PAN fibers, especially when commercial CNTs with high metal content are used.^45,46^ This study presents a cost-effective and sustainable method for synthesizing multiwalled carbon nanotubes (MWCNTs) from recycled plastic waste, directly addressing both environmental concerns and material performance limitations. By using stainless steel (SS316) substrates and catalytic CVD, the process yields high-quality MWCNTs with reduced residual metal content, overcoming one of the major drawbacks of commercial CNTs. This study also successfully fabricated high-strength PAN–MWCNT composite fibers from waste-derived CNTs using wet spinning, achieving superior tensile properties compared to fibers reinforced with commercial CNTs. A systematic investigation of CNT morphology, catalytic purity, and composite fiber crystallinity was conducted, revealing that the waste plastics-derived MWCNTs not only improve fiber strength but also offer broader applicability in structural, energy, electronic, and smart textile applications. This work establishes a scalable, circular approach to convert plastic waste into value-added carbon nanomaterials, advancing the fields of nanocomposite manufacturing and sustainable materials engineering.

## 2 Results and Discussion

### 2.1 Overview of the Project

Figure 1 illustrates a novel closed-loop process for converting postconsumer plastic waste into high-performance PAN–MWCNT composite fibers, addressing the global challenges of plastic pollution, carbon circularity, and the high cost of conventional nanomaterials. This integrated system not only provides a pathway for valorization of pyrolysis products from plastic wastes but also enables the direct production of advanced functional fibers using industry-compatible wet-spinning techniques, setting a precedent for circular economy approaches in materials manufacturing. The process begins with the synthesis of MWCNTs from postconsumer plastics via catalytic CVD, as shown in Figure 1a. A two-stage quartz tube system is employed, where Furnace 1 is used for low-density polyethylene (LDPE) pyrolysis to generate hydrocarbon-rich vapors and hydrogen. Furnace 1 was operated at 800 °C to maximize the yield of gaseous pyrolysis products.^47^ Pyrolyzate gases of polyethylene at a furnace temperature of 800 °C have been reported by Zhuo et al. to include methane, ethane, ethylene, propane, propylene, acetylene, butene, butadiene, ethylacetylene, benzene and hydrogen, in good agreement with the findings of Conesa et al.^28,47^ Furnace 2 was also operated at a temperature of 800 °C to facilitate CNT growth on pretreated SS316 wire mesh catalysts (the composition detailed in Table S1), following the recommendation of Zhuo et al.^28,48^ The pyrolysis gases pass through a Venturi mixer for uniform mixing and a high-temperature silicon carbide honeycomb filter (manufactured by Ibiden) to remove particulates larger than 1 μm, ensuring a clean gas stream for CNT synthesis.

**Figure 1:**
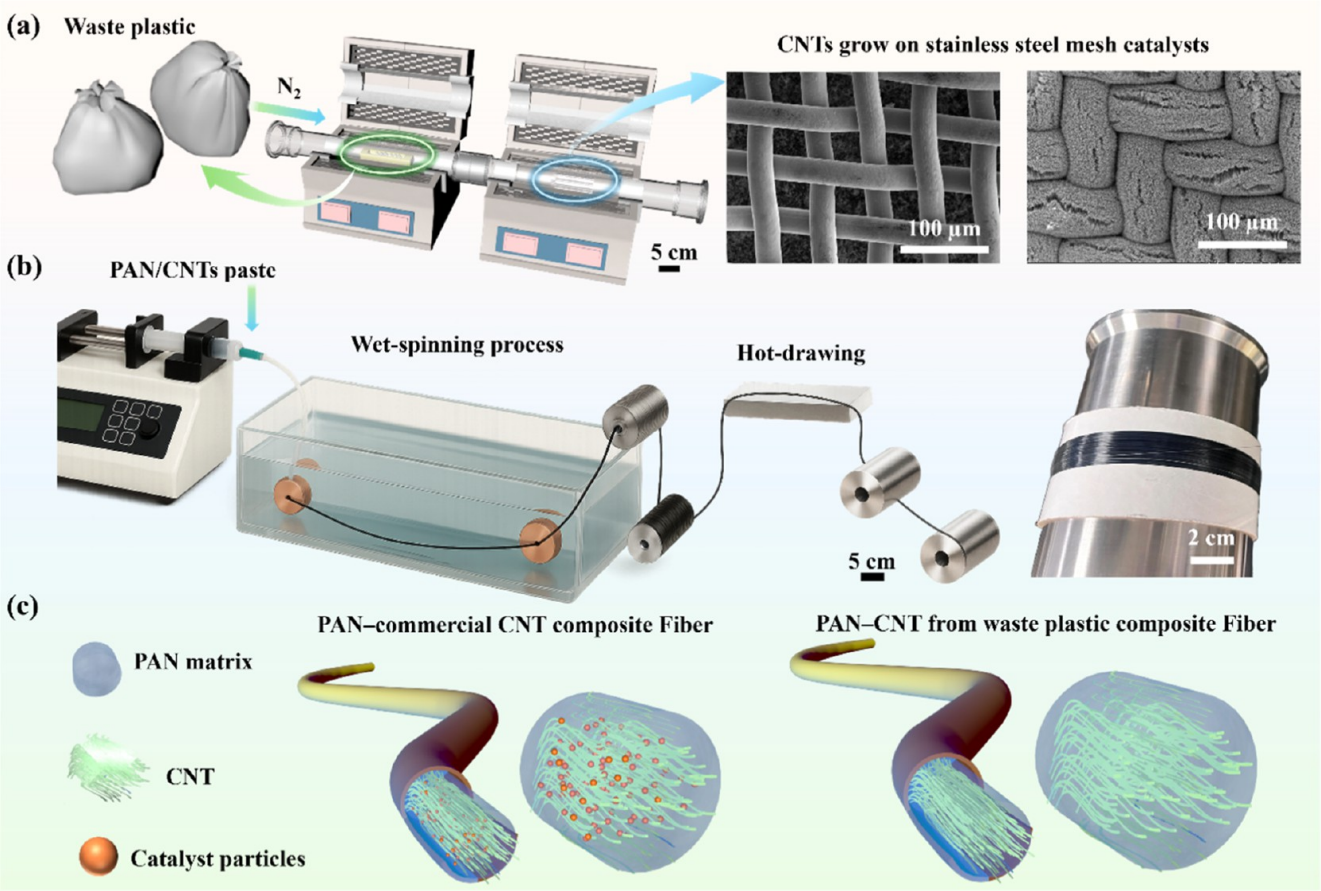
Schematic overview of the transformation of low-value waste polymers into high-value CNTs incorporated into PAN-based composite fibers. (a) Waste plastics-based pyrolysis of postconsumer LDPE waste generating hydrocarbon pyrolyzates and hydrogen for CNT synthesis. (b) The setup for PAN–CNT composite fiber formation enables fiber production via wet spinning followed by hot drawing. The photograph presents the final PAN–CNT fiber. (c) Comparison between PAN–commercial CNT composite fibers and PAN–CNT composite fibers derived from waste plastics, highlighting the influences from catalyst residue and CNT impacts on PAN-based fibers.

A custom catalyst pretreatment protocol was applied to enhance CNT yield and quality, as outlined in our group’s previous work.^28,48^ SS316 meshes are cut into 7 cm × 3 cm rectangles (Figure S1a), ultrasonically cleaned in isopropyl alcohol, and subjected to acid etching (38% HCl mixed with deionized water) to remove surface oxides and increase surface roughness. The meshes are then rapidly heated to 800 °C in air, followed by quenching to stabilize catalytic activity (Figure S1b). After CNT growth via CVD (Figure S1c), the CNTs are detached from the substrate by sonication in ethanol for 120 min, facilitating efficient CNT recovery for subsequent composite fiber fabrication. Importantly, this system incorporates catalyst reuse, significantly improving process sustainability and cost efficiency. The catalytic substrates are reconditioned through an air-cleaning cycle at 800 °C, ultrasonic washing in isopropyl alcohol, and acid pickling to remove any residual CNTs and restore surface activation.^15,28,48^ This multicycle reusability of SS316 catalysts reduces both material consumption and processing waste, advancing the environmental and economic viability of CNT production.^49^ The yield of carbon nanotubes was evaluated in two ways: based on the weight of the catalytic substrate (Y_c_) and based on the weight of the polymer feedstock (Y_p_), both measured while the CNTs remained attached to the substrate. The final yield (Y_final_) was determined after the CNTs were separated from the substrate and collected, calculated relative to the weight of the polymer feedstock. Figure S2 presents the CNT yields (Y_c_, Y_p_, and Y_final_) after each use of SS-316 mesh 400 substrates following treatment. Figure S2 reproduced from the author’s Master’s thesis.^49^ The MWCNTs synthesized from waste plastics using this method exhibit bamboo-like morphology and significantly reduced catalyst contamination, as confirmed by transmission electron microscopy (TEM) and energy-dispersive X-ray (EDX) element mapping analyses discussed in following sections. Compared to commercially available CNTs, these waste-derived MWCNTs possess cleaner surfaces and lower residual metal content, addressing one of the most pressing issues in CNT composite manufacturing: the detrimental effects of metal catalyst residues on composite performance (e.g., act as stress concentrators and reduce load transfer). This improvement ensures better dispersion and stronger CNT–matrix interfacial bonding, which are critical factors for mechanical reinforcement in polymer composites.

Following CNT synthesis, the MWCNTs are incorporated into PAN matrices to produce composite fibers via wet spinning, as illustrated in Figure 1b.^50,51^ The PAN–MWCNT precursor solution is injected via a syringe pump into a methanol coagulation bath at a controlled rate of 0.3 mL/min, forming as-spun fibers that are collected by take-up rollers operating at 5 m/min. The methanol flow rate, injection rate, and take-up speed are meticulously optimized to ensure uniform fiber morphology, consistent coagulation, and minimal defect formation. Subsequently, the fibers are subjected to hot drawing, a postprocessing step that aligns the PAN molecular chains and densifies the fiber structure. This step is essential for enhancing mechanical performance, as it promotes increased crystallinity and reduced porosity. The photograph of the final PAN–CNT fiber after the final-stage hot drawing process is also presented. As a result, the comparison between PAN fibers reinforced with commercial CNTs and those with waste-derived CNTs, as illustrated in Figure 1c, demonstrates superior stiffness and strength in the fibers incorporating CNTs from waste plastics. Mechanical testing demonstrates that the PAN–MWCNT composite fibers achieve a Young’s modulus of 13.4 GPa and a tensile strength of 941 MPa, far surpassing both neat PAN fibers and PAN composites reinforced with commercial CNTs. This significant improvement is primarily attributed to the low metal catalyst impurity content and high-quality structural features of the waste-derived MWCNTs.

In contrast to conventional CNT synthesis routes, which often rely on expensive catalysts and high-purity feedstocks, this approach valorizes low-cost waste streams to produce advanced composite materials, representing significant progress toward carbon-neutral nanomanufacturing. By integrating CNT growth, catalyst regeneration, and fiber fabrication into a closed-loop process, this method exemplifies a circular economy model for advanced materials production, with potential applications in aerospace, textiles, structural reinforcement, and flexible electronics. Beyond improving the CNT synthesis and composite performance, this work addresses the broader environmental and economic challenges associated with both plastic waste accumulation and the high cost of commercial CNTs. Specifically, by converting postconsumer plastics into high-value nanomaterials, this method also provides a sustainable, low-cost alternative to CNTs traditionally sourced from petroleum-derived precursors. More importantly, our system leverages standard industrial wet-spinning and fiber processing techniques, requiring no substantial modifications to existing manufacturing infrastructure, further enhancing its scalability and practical relevance.

### 2.2 CNT Synthesis from Waste Plastics

Figure 2 illustrates the catalytic growth process, mechanisms, and morphological characterization of MWCNTs synthesized from plastic-derived carbon sources via CVD using an Fe-based SS316 wire-mesh catalyst. The schematic in Figure 2a highlights the base-growth mechanism, where hydrocarbon precursors decompose on the catalyst surface, allowing carbon atoms to diffuse through and precipitate as tubular CNT structures.^25,52^ In this process, the iron nanoparticles present in SS316 act as active catalytic sites, facilitating the nucleation and growth of MWCNTs under reductive and hydrocarbon-rich conditions. Unlike tip-growth, where the catalyst detaches and lifts during CNT formation, base-growth involves the catalyst remaining anchored to the substrate, which is advantageous for aligned CNT synthesis and scalable production. The CNT morphology is strongly influenced by parameters such as catalyst composition, temperature, gas flow rate, and reaction time. In this study, using waste plastic as the carbon feedstock leads to the formation of bamboo-like MWCNTs, a structure commonly associated with tip-growth interruptions or fluctuating growth environments. Importantly, this characteristic morphology also gets benefited from the periodic catalyst encapsulation or defect formation during CNT growth, confirming the feasibility of converting plastic waste into high-value CNTs using a reusable Fe-based SS316 catalyst through a controlled CVD process.

**Figure 2:**
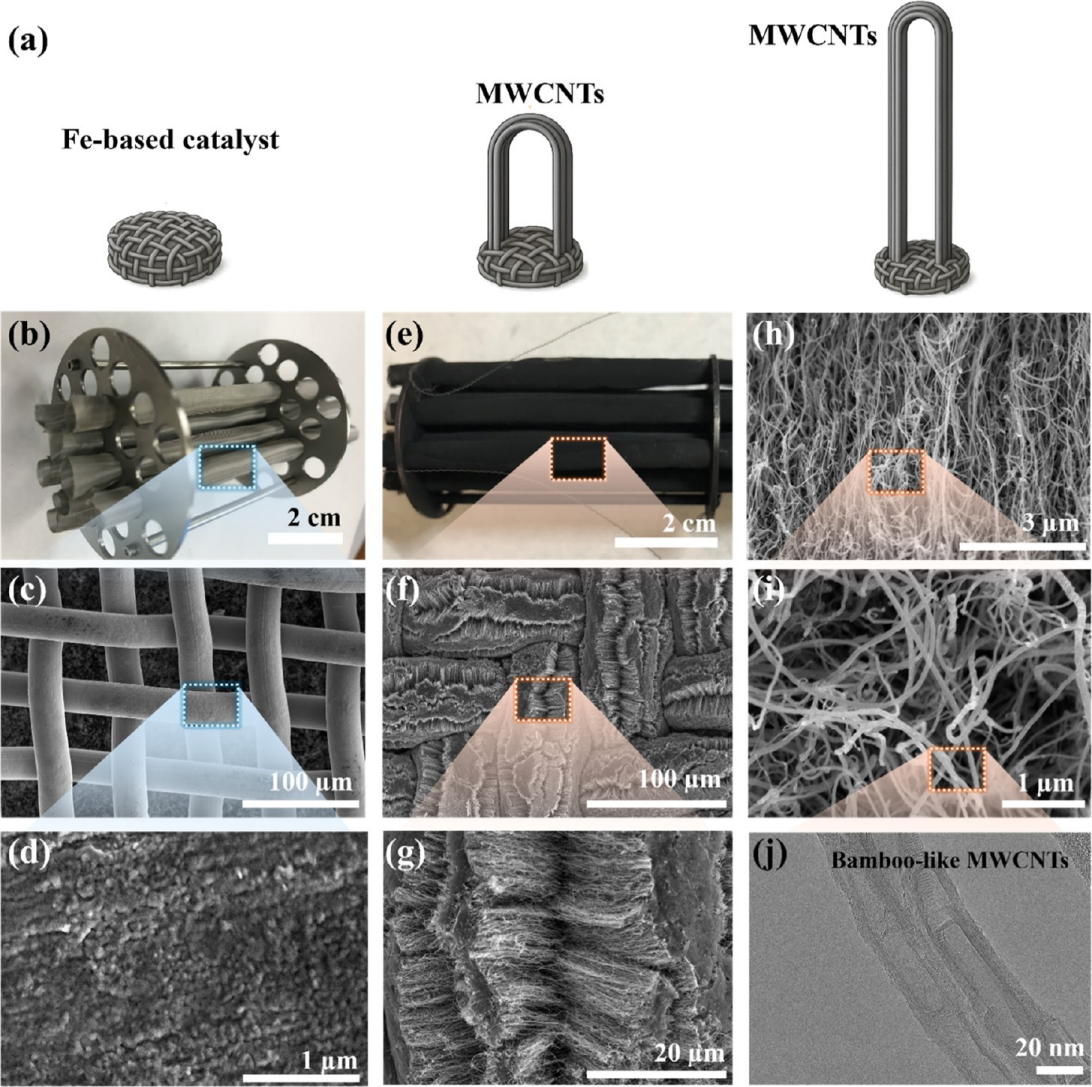
Schematic and morphological characterization of MWCNTs synthesized from plastic waste via CVD using an Fe-based SS316 wire cloth acting as a catalyst (due to Fe/Ni content). (a) Schematic diagram illustrating the CNT growth mechanism on a catalyst surface via the tip-growth or fluctuating conditions, where hydrocarbon precursors produce carbon species that diffuse into and precipitate from catalyst sites to generate MWCNTs. (b–d) Photographic and SEM images of SS316 catalyst before CNT growth, showing surface morphology at progressively higher magnifications. (e–i) Photographic and SEM images of the SS316 catalyst after CVD growth of MWCNTs from plastic-derived carbon feedstocks, revealing extensive CNT formation at multiple scales. (j) High-resolution TEM image highlighting the bamboo-like structure of the resulting MWCNTs, a characteristic morphology often associated with tip-growth or defect-mediated processes.

Figure 2b–d display the photographic and scanning electron microscopy (SEM) images of the SS316 catalyst substrate before CNT growth, highlighting the pretreatment process applied to activate the stainless-steel mesh. The SS316 wire cloth, specified as 400 × 400 mesh with a wire diameter of 29 μm, serves as a fixed catalyst substrate. Pretreatment was performed to disrupt the native chromium oxide (Cr_2_O_3_) layer, keeping the actual catalytic sites from Fe and Ni in SS316, increasing surface roughness and exposing catalytic sites essential for CNT nucleation. SEM images (Figure 2c,d) reveal a fine microstructure with well-defined grain boundaries, where the grain size and surface-exposed Fe/Ni particle grain size formed during reduction correlates with the eventual diameter of the synthesized MWCNTs. Following CVD growth using LDPE as the carbon feedstock, the morphological evolution of the SS316 catalyst is shown in Figure 2e–i. The catalyst wires become densely coated with nanocarbon structures, forming a CNT forest morphology characterized by uniformly separated, long, and smooth MWCNTs with minimal entanglements. The diameters of these fibers vary, even along the same fiber. Higher magnification SEM images (Figure 2h,i) confirm the formation of dense, continuous MWCNT networks, indicating the high catalytic efficiency of SS316 under plastic-derived carbon CVD conditions.

A high-resolution TEM (HRTEM) image in Figure 2j reveals that the resulting MWCNTs exhibit a bamboo-like internal structure, commonly associated with tip-growth modes or fluctuating carbon supply during the base-growth mechanism. Specifically, this morphology may result from fluctuating growth conditions, such as inconsistent hydrocarbon feedstock decomposition or catalyst instability inherent to plastic-derived sources. Bamboo-like MWCNTs can offer distinct advantages, including varying defect density and enhanced surface area, which can be beneficial for applications in energy storage, adsorption, and catalysis. Further examination of SEM images from catalyst reuse experiments (Figure S3) indicate that repeated CVD cycles lead to increased CNT accumulation on both the individual wires and interstitial spaces of the SS316 mesh. Over successive uses, the morphology of the CNTs evolves from uniformly dispersed and well-aligned tubes to entangled globular bundles with shorter lengths and a broader diameter distribution. This suggests that catalyst reuse introduces microstructural changes, potentially due to catalyst site saturation, surface degradation, contamination from previous cycles or carbon encapsulation of Fe/Ni particles, which influence CNT growth behavior, confirming the integration of plastic-derived carbon feedstocks into CNT synthesis not only provides a sustainable materials pathway but also enables the production of CNTs with unique morphologies for advanced applications.

Figure 3 presents TEM images of three types of CNTs, namely, the Figure 3a mixed CNTs consisted of SWNTs and DWNTs, Figure 3b single-walled CNTs (SWCNTs), and Figure 3c MWCNTs, captured at increasing magnifications of 20,000×, 60,000×, and 100,000× to examine their morphological and structural differences in detail. The mixed CNTs (Figure 3a_1_–a_3_), typically synthesized through nonselective or unrefined processes and here a commercial mixture of SWNTs and DWNTs, display a highly entangled and structurally heterogeneous morphology. At higher magnification (Figure 3a_3_), distinct bundling, overlapping, and occasional collapse of tubes are evident. The presence of amorphous carbon residues and metallic nanoparticles (highlighted in yellow) indicates a high level of impurities, which could interfere with CNT dispersion and load transfer when embedded in polymer matrices. This structural irregularity not only limits the efficiency of stress distribution but also hinders interfacial bonding in composite systems. The SWCNTs (Figure 3b_1_–b_3_), although consisting of thinner and potentially more flexible tubes, also exhibit significant bundling and entanglement. The darker spots and uneven texture visible in Figure 3b_2_,b_3_ are likely residual stainless steel catalyst or defects introduced during purification or synthesis. These impurities can act as nucleation sites for crack propagation, which negatively impacts the mechanical integrity of CNT-reinforced composites. Moreover, the strong van der Waals interactions between individual SWCNTs contribute to their natural tendency to bundle, complicating uniform dispersion in solution or matrix systems.

**Figure 3:**
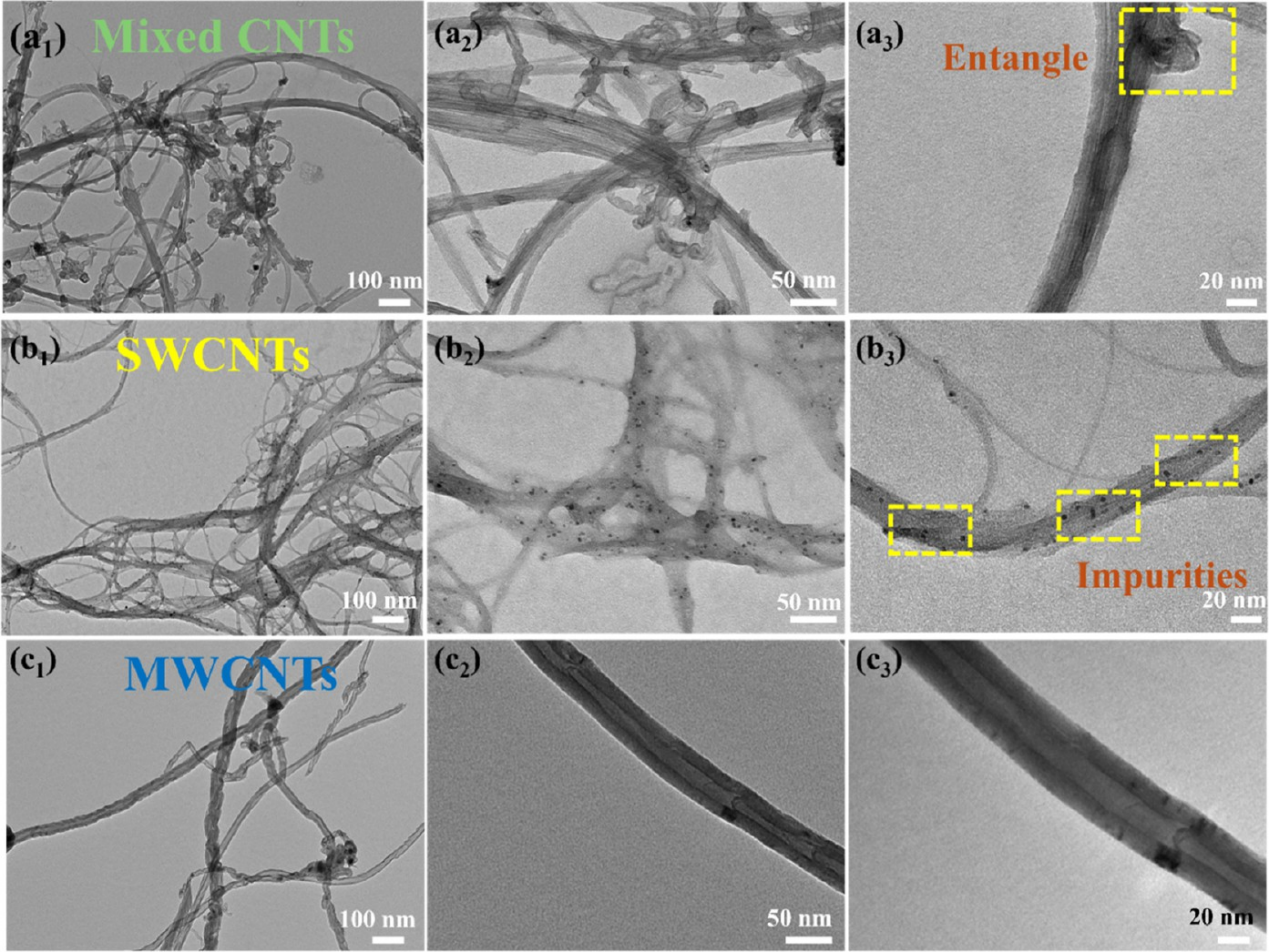
TEM images of three types of CNTs separated from catalyst substrates at increasing magnifications (left to right: 20,000×, 60,000×, and 100,000×). (a_1_–a_3_) Mixed CNTs from commercial sources, showing entangled nanotube structures and occasional agglomerates (highlighted in yellow). (b_1_–b_3_) Commercially sourced SWCNTs, where the presence of impurities is visible as particulate residues on the tube surfaces (highlighted in yellow). (c_1_–c_3_) MWCNTs obtained from plastic-derived carbon, displaying well-defined multiwall structures with fewer surface impurities.

In contrast, the MWCNTs derived from plastic waste from this research (Figure 3c_1_–c_3_) show clearly defined, smooth-walled tubular structures with concentric graphene layers and significantly fewer visible surface contaminants. These tubes appear longer, more isolated, and straighter than those in the other groups, indicating better graphitization and structural integrity. This morphology suggests that MWCNTs, especially those synthesized from waste-based carbon sources, may offer superior mechanical reinforcement potential due to their reduced entanglement and enhanced dispersibility. Such features are particularly advantageous in high-performance composite applications, provided appropriate surface functionalization is applied to optimize matrix interaction. Further insights are provided by Figure S4, which shows TEM images of MWCNTs synthesized after the second (Figure S4a) and fourth (Figure S4b) reuse cycles of the SS316 catalyst. The comparison indicates a progressive degradation of CNT morphology with increasing catalyst reuse. In the early cycles, CNTs are relatively uniform and well structured. By the fourth reuse, however, pronounced changes emerge, including greater curvature and entanglement, shorter tube lengths, and rougher, less defined surfaces. Additionally, as the number of reuse cycles increases, diverse morphologies appear, such as coiled, twisted, and roped tubes, accompanied by intensified surface ruggedness. This evolution marks a clear transition from uniformly dispersed, straight CNTs to more entangled, globular bundles. As the active Fe/Ni clusters coarsen and lose surface accessibility, the catalytic efficiency for carbon precursor decomposition declines, leading to slower growth rates and uneven carbon deposition. The reduced catalytic uniformity further promotes localized overgrowth and tube entanglement, intensifying the morphological disorder. These transformations are consistent with catalyst evolution under repeated high temperature CVD and carbon rich feed, including the deactivation and sintering of active sites on SS316, carbon encapsulation of Fe/Ni nanoparticles, phase segregation, and detachment of loose catalyst particles. This microstructural evolution correlates directly with declining CNT quality. Coiled and twisted morphologies are associated with elevated defect densities and wall discontinuities. Reduced tube lengths limit load transfer and percolation. Impurity accumulation, particularly residual catalyst trapped within entangled bundles, further degrades purity. Together, these factors lower CNT crystallinity and increase residual metal content, which in turn diminishes the mechanical performance of PAN–CNT composite fibers.

These observations have highlighted the importance of both CNT source and catalyst longevity in determining nanotube quality. As compared to commercial SWNTs or SWNTs/DWNTs, the MWCNTs derived from plastic waste not only exhibit competitive morphological characteristics (i.e., fewer impurities, better graphitization, or longer tube lengths) compared to commercial sources but also demonstrate potential for sustainable nanomaterial production. However, attention must be given to catalyst regeneration strategies and process optimization (e.g., periodic acid cleaning, controlled oxidation, or thermal treatments to restore active catalytic sites and maintain CNT quality over cycles) to maintain CNT quality during extended catalyst use. These findings further reinforce that structural purity, alignment, and length uniformity are key parameters that govern the reinforcement efficiency of CNTs in composite systems. To further evaluate the impact of catalyst reuse on CNT quality, thermogravimetric analysis (TGA) was conducted to examine the effects of repeated catalyst recycling. As shown in Figure S5, the residual weight progressively increased with each reuse cycle, indicating the accumulation of iron and other catalyst residues within the CNT products. These residues, along with structural defects generated during multiple growth cycles, are believed to catalyze premature cyclization and degradation of PAN during stabilization and carbonization. Consequently, this phenomenon leads to a gradual decline in the mechanical properties of the composite fibers with successive catalyst reuse. Specifically, repeated reuse causes the catalytic substrate to become increasingly fragile, resulting in the detachment of loose catalyst particles and the formation of CNTs with higher defect densities and elevated residual metal content. As shown in Figure S5, the residue content of CNTs obtained after the sixth reuse reaches approximately 50%, compared to only 12–14% from the initial use, clearly demonstrating a significant increase in metallic impurities. Raman spectroscopy was also employed to evaluate the quality of the CNTs, as shown in Figure S6. The purity of the collected MWCNTs was calculated and is summarized in Table S2. The highest measured purity reached 74%, with good consistency among the three Raman intensity ratios (ID/IG, IG′/IG, and IG′/ID) observed only during the initial use of both meshes. The calculated purity values indicate the presence of certain structural defects and/or nontubular carbon species within the nanomaterials. They may also reflect the existence of amorphous carbon and residual metal particles.

To further elucidate the structural characteristics and purity of the synthesized CNTs, HRTEM combined with EDX elemental mapping was performed, as shown in Figure 4. This analysis provides detailed insight into both the morphology of the CNTs and the distribution of residual catalyst detached from the wire screen. These are critical considerations for nanocomposite and electronic applications where metal impurities can significantly impact performance and biocompatibility. Figure 4a_1_–a_4_ present the HRTEM image and corresponding EDX maps of mixed CNTs, revealing a network of entangled tubes with numerous bright-contrast regions corresponding to metal nanoparticles embedded in the CNT matrix. The EDX elemental mapping shows a uniform distribution of carbon (green) across the sample, confirming the nanotube framework. However, concentrated cobalt (Co) signals (yellow) are observed in localized clusters, directly correlating with the metal nanoparticles in the HRTEM image. This indicates that Co-based catalysts, commonly used for CNT synthesis due to their high catalytic efficiency in decomposing hydrocarbons, remain embedded in the final product. Additional EDX spectra (Figure S7) confirm this finding, showing strong peaks for Co alongside carbon, with minor signals for molybdenum (Mo) and chromium (Cr), likely originating from alloy components in the catalyst or reactor materials.

**Figure 4:**
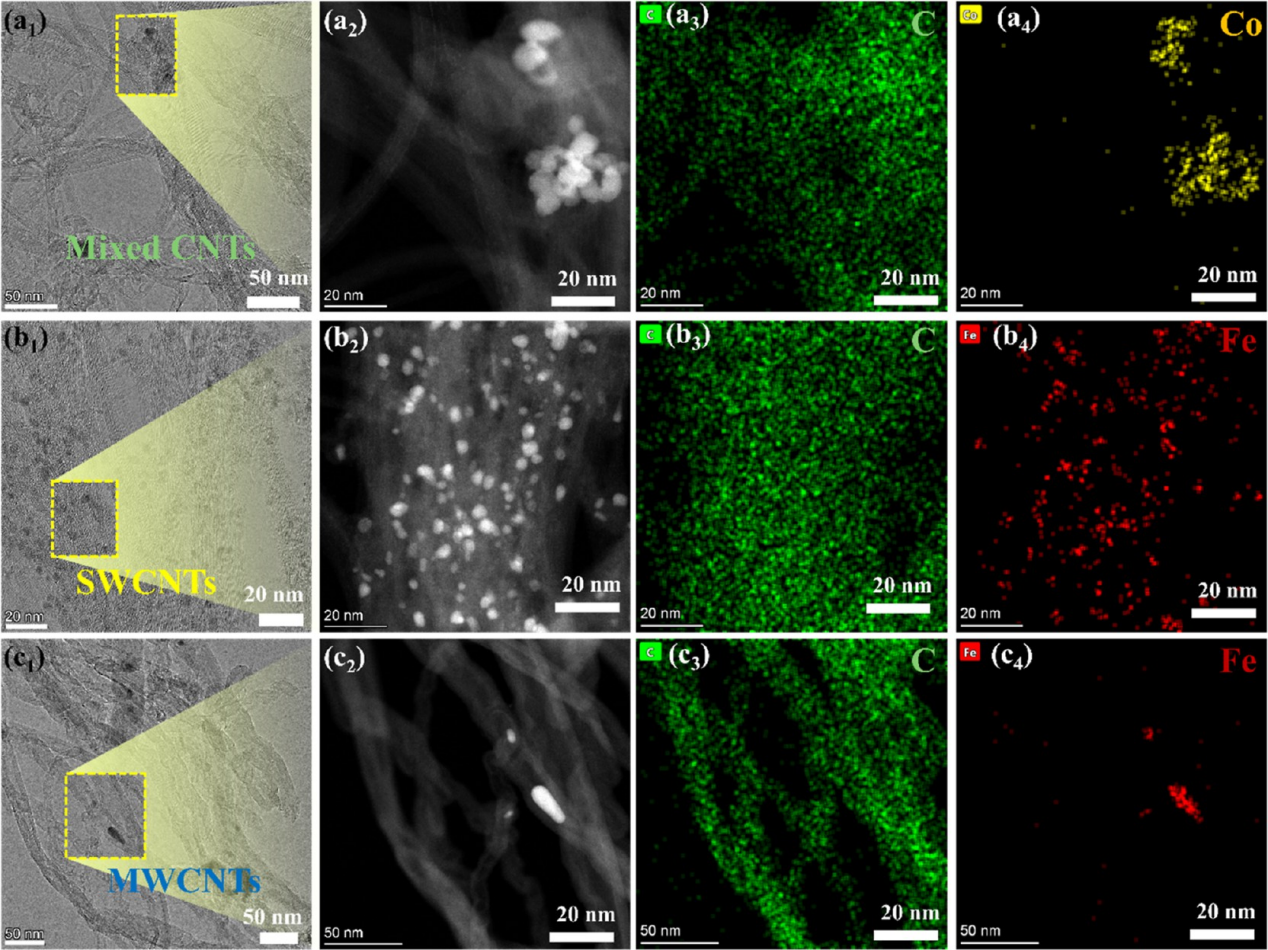
High-resolution TEM (HRTEM) images and corresponding EDX elemental maps for carbon (C), cobalt (Co), and iron (Fe) in three CNT types. (a_1_–a_4_) Mixed CNTs (commercial SWCNT/DWCNT blend) showing carbon (green) and cobalt (yellow) distributions, indicating significant residual Co catalyst particles. (b_1_–b_4_) Commercial SWCNTs with carbon (green) and dispersed iron (red), highlighting Fe-based catalyst impurities commonly retained after synthesis. (c_1_–c_4_) Waste plastic–derived MWCNTs with carbon (green) and sparse localized iron (red), confirming markedly lower residual metal content compared to commercial CNTs. These EDX results aligns with previous observations that the superior purity of MWCNTs synthesized via the plastic-to-CNT process may potentially support their improved performance in PAN composite fibers.

In Figure 4b_1_–b_4_, the HRTEM image and EDX maps of SWCNTs further highlight the issue of residual catalyst contamination. The carbon signal remains uniform throughout the sample, but iron (Fe) is observed in discrete, localized clusters (red). This is consistent with the use of Fe-based catalysts in SWCNT production, where Fe nanoparticles often act as nucleation sites for tube growth. However, Fe particles are notoriously difficult to remove from SWCNTs due to their small size and strong adhesion to carbon surfaces. The EDX spectrum in Figure S8 confirms this, showing prominent Fe peaks along with carbon. Minor copper peaks are also detected, originating from the TEM support grid. These findings emphasize the necessity of rigorous postsynthesis purification to mitigate the negative effects of metal impurities on CNT applications, particularly in fields like electronics or biomedical engineering where residual metals can alter conductivity, toxicity, or surface chemistry.

In contrast, Figure 4c_1_–c_4_ shows HRTEM images and EDX maps of our MWCNTs derived from plastic waste. The MWCNTs display well-defined concentric walls and a smooth, tubular morphology with a uniform carbon distribution (green) and minimal Fe signals (red) in the EDX maps. The corresponding EDX spectrum in Figure S9 supports this observation, showing a dominant carbon peak with only weak iron signals. Minor copper peaks again arise from the TEM grid. The low iron content in the MWCNTs suggests more effective catalyst encapsulation, removal, or passivation during growth, possibly due to the synthesis conditions or the nature of the plastic-derived carbon feedstock. Studies have shown that MWCNTs often retain fewer catalyst residues than SWCNTs due to their larger diameters and the ability of metal nanoparticles to remain anchored at the substrate during base-growth processes. Comparing the three samples, the mixed CNTs and SWCNTs usually typical from commercial sources exhibit significantly higher levels of catalyst residue, as evidenced by the strong Co–Kα/β and Fe–Kα/β peaks in the EDX spectra (Figures S7–S9). The MWCNTs from waste plastics, however, display much lower residual metal content, indicating a higher degree of structural purity. This is advantageous for applications where the presence of metal impurities could compromise electrical, mechanical, or biocompatibility properties. For example, in polymer composites we demonstrate in following sections, excess catalyst can act as stress concentrators, reducing tensile strength, while in electronics, metal residues can cause unintentional doping or interfere with charge transport.

### 2.3 CNT Reinforcement in Composite Fibers

Figure 5 presents a comprehensive analysis of the structural and thermal properties of PAN–CNT composite fibers, synthesized using CNTs derived from various feedstocks and catalyst reuse stages. The wet spinning process, shown in Figure 5a, was employed to fabricate the PAN–CNT composite fibers. Following spinning, the fibers were coagulated in methanol for 36 h to solidify the structure and remove residual solvent. The fibers were then subjected to a hot drawing process using a heated plate (10 in. × 1 in.), which aligns the molecular chains, reduces porosity, and improves mechanical properties such as tenacity and stiffness. This postdrawing treatment is critical for enhancing the load-bearing capacity of the fibers by promoting molecular orientation and densification. The drawn fibers are shown in Figure 5b, and the SEM image in Figure 5c confirms a smooth and uniform surface morphology, indicative of successful processing. Further insights into the cross-sectional structure of these fibers are provided in Figure S10, which shows optical microscopy images comparing fiber diameters after three drawing stages. The PAN–CNT fibers fabricated using the initial catalyst use exhibited an average diameter of 46 μm, while fibers produced after the second and fourth catalyst reuse cycles showed progressively larger diameters of 60 and 70 μm, respectively. This increase in diameter with catalyst reuse likely reflects changes in CNT morphology, including reduced quality, shorter tube lengths, and increased agglomeration, which may inhibit effective chain alignment during drawing.

**Figure 5:**
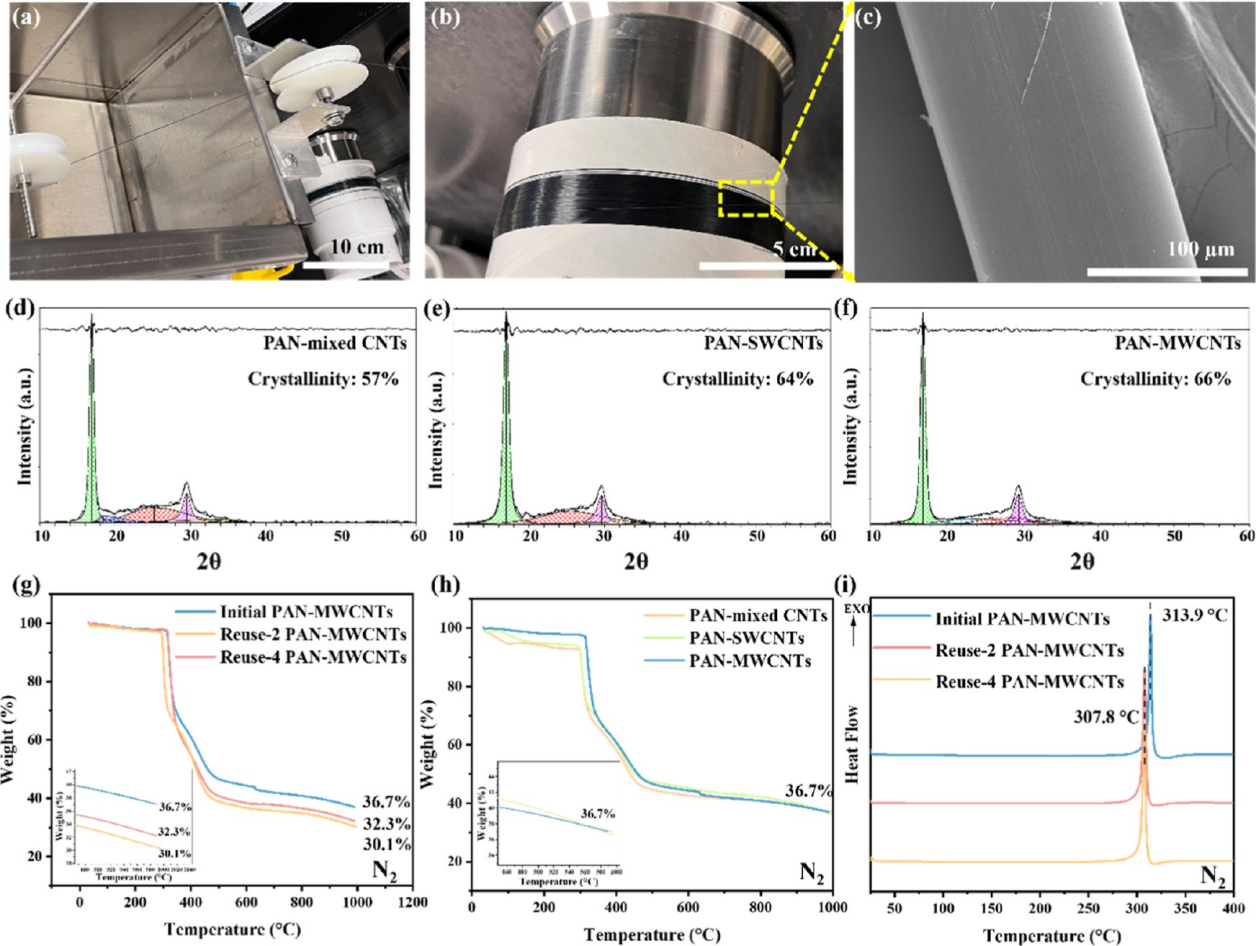
Structural and thermal characterization of PAN–CNT composite fibers. (a) Image of fiber formation during the wet spinning process. (b) PAN–CNT composite fibers after hot drawing. (c) SEM image showing the surface morphology of a PAN–CNT fiber postprocessing. (d–f) XRD patterns and deconvoluted peaks of PAN–CNT composite fibers incorporating different CNT types: (d) PAN with mixed CNTs (crystallinity: 57%), (e) PAN with SWCNTs (crystallinity: 64%), and (f) PAN with MWCNTs (crystallinity: 66%). (g–i) Thermal analysis of PAN–CNT composite fibers using SDT and DSC. (g) SDT curve showing weight loss profiles for different CNT composites under nitrogen. (h) SDT results for PAN–MWCNT composites after multiple catalyst reuse cycles, highlighting differences in residual mass. (i) DSC curves of PAN–MWCNT composites at various catalyst reuse stages, indicating changes in thermal transitions and melting behavior under inert atmosphere.

The X-ray diffraction (XRD) patterns in Figure 5d–f reveal the crystallinity evolution of the PAN–CNT composite fibers after final drawing. All samples exhibit characteristic crystalline peaks at 2θ ≈ 17° and 30°, corresponding to the (110) and (020) planes of PAN. The degree of crystallinity, calculated by integrating the deconvoluted peak areas, is 57% for mixed CNTs (Figure 5d), 64% for SWCNTs (Figure 5e), and 66% for MWCNTs (Figure 5f). These findings are consistent with previous studies showing that CNTs can act as nucleating agents to promote PAN chain alignment during fiber formation. The higher crystallinity observed in the MWCNT-containing fibers suggests that multiwalled structures provide superior nucleation sites due to their larger surface area and concentric graphitic walls. Increased crystallinity is directly linked to improved mechanical properties, as it enhances load transfer efficiency and resistance to deformation in composite fibers.

Thermal stability was assessed using simultaneous differential thermal analysis (SDT) and differential scanning calorimetry (DSC), shown in Figure 5g–i. The SDT curves in Figure 5g show that all PAN–CNT composites undergo a major weight loss between 300–400 °C, corresponding to the cyclization and degradation of PAN. The total residual mass was highest in the PAN–MWCNT composites (36.7%), suggesting that MWCNTs provide enhanced thermal stabilization through char formation, consistent with their higher crystallinity and graphitization. PAN–SWCNT and mixed CNT composites exhibited slightly lower residual mass, indicating less thermal stability, likely due to differences in CNT dispersion, aspect ratio, and purity. Figure 5h explores the impact of catalyst reuse on thermal stability. Composites incorporating CNTs from the initial catalyst use retained 36.7% of their mass, while those using CNTs from the second and fourth reuse cycles retained 32.3% and 30.1%, respectively. This trend suggests that repeated catalyst use leads to CNTs with higher defect densities, increased amorphous carbon content, and reduced tube lengths, which compromise the thermal stability of the composites. These findings align with previous reports indicating that catalyst aging diminishes CNT quality, resulting in materials with lower oxidation resistance and structural integrity.

The DSC curves in Figure 5i provide further confirmation of the relationship between CNT quality and thermal behavior. All samples display a single exothermic peak associated with the cyclization of PAN, a crucial step in CF precursor conversion. The initial-use CNT composite shows a peak at 313.9 °C, while the second and fourth reuse composites exhibit peaks at 307.8 °C, indicating a shift to lower transition temperatures. This reduction reflects the presence of CNT defects and catalyst residues introduced during extended reuse, which may catalyze premature cyclization or degradation reactions. Additionally, lower-quality CNTs may disrupt the heat conduction pathways, leading to heterogeneous thermal behavior within the fiber.

Mechanical performance, including Young’s modulus and tensile strength, is critical for the application of fiber composites in sectors such as aerospace, automotive, and flexible electronics. High modulus provides stiffness and dimensional stability, while high tensile strength ensures the material can withstand applied loads without fracture. In fiber-reinforced composites, achieving a balance of these properties is essential for producing lightweight, durable, and high-strength materials. Figure 6a,b present a comparative analysis of the mechanical properties of PAN–CNT composite fibers reinforced with three different CNT types: mixed CNTs, SWCNTs, and MWCNTs. The data clearly show that CNT type plays a significant role in determining reinforcement efficiency. For Young’s modulus (Figure 6a), all CNT-reinforced PAN fibers outperformed the control PAN fibers (8.3 GPa). The highest modulus was achieved with MWCNT reinforcement (13.4 GPa), followed by SWCNTs (13.0 GPa) and mixed CNTs (12.8 GPa). This enhancement can be attributed to the rigid multiwalled structures of MWCNTs, which act as efficient load-bearing reinforcements, facilitating stress transfer through the PAN matrix. Higher stiffness also correlates with improved crystallinity and molecular alignment, both of which were confirmed in the XRD results discussed previously (Figure 5).

**Figure 6:**
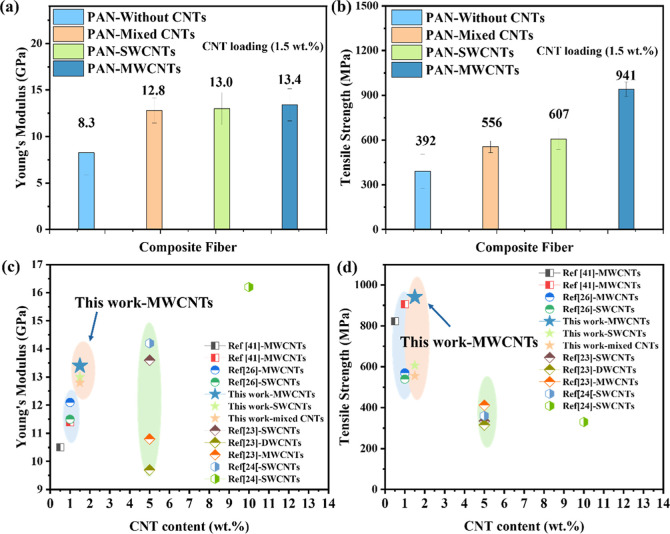
Mechanical performance comparison of PAN–CNT composite fibers incorporating different CNT types. (a) Young’s modulus and (b) tensile strength of PAN composite fibers reinforced with mixed CNTs, SWCNTs, and MWCNTs, compared to control PAN fibers without CNTs. (c,d) Benchmark comparison of this work with literature data. (c) Young’s modulus and (d) tensile strength of PAN–CNT composite fibers in this study versus previously reported CNT-reinforced PAN fibers, highlighting the superior performance of waste-derived MWCNT composites in both stiffness and strength.

In terms of tensile strength (Figure 6b), a similar trend is observed. PAN–MWCNT fibers achieved the highest tensile strength at 941 MPa, significantly exceeding that of SWCNT (607 MPa) and mixed CNT composites (556 MPa). In contrast, pristine PAN fibers exhibited a much lower tensile strength of 392 MPa. The superior tensile strength of the MWCNT-reinforced fibers can be attributed to better dispersion, reduced entanglement, and fewer structural defects in the MWCNTs, which enhance interfacial bonding and minimize stress concentration points within the composite. The larger diameters and more stable tubular structures of MWCNTs also prevent collapse during processing, further supporting mechanical load transfer.

The structure–property relationship of PAN–CNT composites is governed by multiple factors, including crystallinity, molecular alignment, and interfacial bonding quality. Increased crystallinity limits chain mobility, thereby enhancing fiber stiffness (Figure 5). Additionally, reducing catalyst residues and removing impurities (Figures 3 and 4) are essential for achieving consistent mechanical performance, as metallic contaminants can act as defects, leading to premature failure under load. In this work, the use of SS316 catalyst in a base-growth CVD process, combined with sonication and acid purification of CNTs, effectively minimized residual catalyst content, as confirmed by TEM and EDX analyses. This improvement in purity directly contributed to the observed mechanical enhancements.

Further investigation into catalyst reuse effects revealed a gradual decrease in mechanical properties with each successive catalyst cycle. Figure S11a,b show a clear reduction in both modulus and strength as the number of catalyst reuses increases, suggesting that catalyst degradation (e.g., metal particle sintering, surface oxidation, or carbon encapsulation) leads to CNTs with higher defect densities, shorter tube lengths, and increased impurity levels. To mitigate this, an acid purification process was applied following the method of Edwards,^53^ using 38% HCl sonication to remove magnetic impurities and metal residues. Posttreatment, the CNTs were filtered and rinsed to neutral pH, and visual comparison in Figure S12 confirms the effectiveness of this process. The acid-treated CNTs demonstrated significantly improved dispersion stability, leading to better composite fiber quality. The mechanical improvements after acid treatment are shown in Figure S11c,d, where both modulus and strength increase following impurity removal. This highlights the detrimental impact of catalyst residues on mechanical performance and underscores the importance of postsynthesis purification for CNT-based composites. Moreover, the effect of nitrogen gas flow rate during CNT synthesis was explored (Figure S13). SEM images revealed that lower flow rates (0.1 L/min) produced denser, more uniform CNT networks compared to higher flow rates (2 L/min), which yielded sparser, more defective structures. Correspondingly, composite fibers fabricated using CNTs grown under low-flow conditions exhibited superior mechanical properties, emphasizing that CNT growth conditions (i.e., flow rate, catalyst reuse) directly impact the quality and performance of final fiber composites (i.e., alignment, defect density, diameter distribution). In the future, catalyst regeneration strategies need to be optimized to sustain CNT quality over multiple reuse cycles. Specifically, we plan to investigate periodic acid cleaning and thermal treatments to remove accumulated impurities, restore catalytic activity, and reduce defect formation during CNT growth. These approaches aim to maintain consistent CNT morphology and purity, thereby improving composite performance and ensuring the long-term viability of the catalyst reuse process. This future work will provide a more sustainable and efficient pathway for CNT production.

Figure 6c,d benchmark the mechanical performance of the PAN–CNT fibers developed in this study against previously reported CNT-reinforced PAN fibers. The data clearly demonstrate that the waste-derived MWCNTs used here outperform previously reported CNT composites in both stiffness and strength, as detailed in Table S2. The composite fibers exhibited improved PAN orientation and crystallite size, with CNTs showing much higher alignment than the PAN matrix. Low-strain property enhancements were linked to PAN–CNT interactions, while high-strain improvements were partially due to CNT length. These effects were closely related to CNT surface area and morphology.^23^ However, previous studies have overlooked the impact of catalyst residue. In this work, the highest tensile strength was achieved, attributed to significantly reduced catalyst residue. The PAN–MWCNT fibers produced in this work not only achieved higher mechanical properties but also offer a sustainable and cost-effective alternative to commercially sourced CNTs. While commercial SWCNTs can cost up to $900 per gram, the waste-derived CNTs used in this study significantly reduce raw material costs, aligning with green manufacturing and circular economy principles. This approach not only improves material performance but also contributes to environmental sustainability by upcycling plastic waste into high-value nanocomposites. To verify the effectiveness of the MWCNT dispersion process and ensure that no large aggregates remained, which could lead to defects and nonuniform properties in the composite fibers, the PAN matrix was examined using FIB–TEM. No obvious MWCNT agglomeration was observed, as shown in Figure S14. The MWCNTs are well dispersed within the PAN matrix, indicating that the dispersion and mixing procedures were effective. Uniform MWCNT distribution was achieved through a combination of solvent-assisted dispersion and mechanical mixing, as detailed in our previous work.^51^ This homogeneous dispersion is crucial for effective load transfer and consistent microstructural properties within the composite fibers, ultimately contributing to improved mechanical performance. Elongation at break is also an important factor in evaluating mechanical performance. As shown in Figure S15, the PAN with upcycled MWCNTs sample exhibits an elongation at break of around 17%, compared to approximately 12% for the PAN with commercial mixed CNTs sample, indicating a noticeably higher capacity for deformation before failure. This enhancement demonstrates the superior extensibility and toughness of the upcycled MWCNT-reinforced fibers.

## 3 Conclusions

This study presents a scalable and sustainable method for converting recycled plastic waste into high-quality MWCNTs through chemical CVD and employing them as effective reinforcements in PAN composite fibers. The waste-derived MWCNTs exhibit superior morphology, lower catalyst residue, and higher structural purity compared to commercial CNTs. When incorporated into PAN fibers via wet spinning and hot drawing, these MWCNTs significantly enhance mechanical properties, achieving a Young’s modulus of 13.4 GPa and a tensile strength of 941 MPa. Postsynthesis acid treatment effectively restores CNT quality after catalyst reuse, further improving composite performance. While this work represents a significant step forward, certain limitations remain. Catalyst aging and bamboo-like internal structures indicate that growth conditions can vary and affect CNT quality. Future efforts will focus on optimizing catalyst regeneration and growth control to achieve consistent high performance over multiple reuse cycles. Overall, this study establishes a circular, low-cost pathway for producing high-performance PAN–CNT fibers and advances sustainable approaches for composite manufacturing.

## 4 Experimental Section

### 4.1 Materials

The PAN used here is a random copolymer containing 0.5 wt % methacrylic acid, with a molecular weight of approximately 230 kg/mol and a density of 1.18 g/cm^3^, supplied by Goodfellow Co. The solvent used in this study is dimethylformamide (DMF), from Fisher Chemical (certified ACS, 99.9%) and used without further purification. The mixed CNTs used in this study consisted of a blend of SWCNTs and DWCNTs, sourced from Cheaptubes Co. These nanotubes featured outer diameters ranging from 1 to 4 nm, inner diameters between 0.8 and 1.6 nm, lengths ranging from 5 to 30 μm, and a density of 1.8 g/cm^3^. The SWNTs used in this study were HiPco-purified and obtained from NanoIntegris, Inc. They contained less than 15 wt % residual catalyst impurities, with diameters ranging from 0.8 to 1.2 nm and lengths between 0.1 and 1 μm. The most common postconsumer recycled polymer, LDPE, was used as the carbon feedstock. SS316 catalyst wire cloth (with the composition detailed in Table S1) was purchased from The Cleveland Wire Cloth & Mfg. Co. Deionized water (DI) was employed as a nonsolvent.

### 4.2 Characterization

#### 4.2.1 CVD Synthesis of MWNTs

The MWCNTs synthesis process described in this study involves: (1) pretreatment of catalytic substrates, (2) CNT growth on and separation from the substrates, and (3) retreatment of the substrates for subsequent growth. In step (1), all SS 316 wire cloths were cut into rectangular pieces measuring 7 cm × 3 cm, as shown in Figure S1a. They were initially cleaned with isopropyl alcohol in an ultrasonic bath, followed by pretreatment steps adapted from Panahi et al., with modifications.^11^ Subsequently, the samples were etched in an acid solution (60 mL of 38% hydrochloric acid (HCl) mixed with 40 mL of deionized water) for 10 min. After drying, they were rolled and placed in a holder, as shown in Figure S1b. The samples were then heated in air at 800 °C for 1 min, followed by rapid air quenching. In step (2), a custom-designed two-stage quartz tube was installed across two electrically heated muffle furnaces, as shown in Figure 1a, and manufactured by HEVI-DUTY ELECTRIC Co. Furnace 1 was designated for LDPE feedstock pyrolysis, while Furnace 2 was used for CNT synthesis. Initially, both furnaces were preheated, and nitrogen carrier gas was introduced through the quartz tube at a flow rate of 0.1 L/min. Once both furnaces reached 800 °C, the pretreated substrates were inserted into Furnace 2, and simultaneously, an alumina boat loaded with postconsumer PE feedstocks was placed into the quartz tube in Furnace 1.^15^ The resulting pyrolysis gases, comprising gaseous hydrocarbons, hydrogen, and trace amounts of tars, then passed through a venturi for enhanced mixing, followed by a high-temperature silicon carbide honeycomb filter (Ibiden) to remove any remaining liquid or solid particles larger than 1 μm. CNT growth on the catalytic substrates, as shown in Figure S1c, began at this stage and continued until no pyrolysis aerosols were detected at the outlet of the quartz tube. Upon completion, the CNT-coated substrates were immersed in ethyl alcohol and sonicated for 120 min to detach the CNTs for the subsequent synthesis of PAN–CNT composite fibers. In step (3), the retreatment of the substrates involved three consecutive processes.^49^ First, the substrates were air-cleaned in an 800 °C furnace to ensure complete removal of any residual CNTs. Second, they were sonicated in isopropyl alcohol for 10 min to achieve further cleaning. Finally, acid pickling in a mixture of 60 mL of 38% HCl and 40 mL of deionized water was carried out to increase surface roughness and remove iron oxides. After retreatment, the substrates were reused for the next cycle of CNT growth.

#### 4.2.2 Characterization of CNTs

SEM, a Hitachi S-4800 SEM, with parameter settings of 3 kV accelerating voltage, 10 μA of beam current and 8 mm working distance, was used to observe the CNTs growth on the substrate. A JEOL1010 TEM was used to characterize the morphology and structural properties of CNTs. HRTEM and EDX mapping were performed using a Cs-corrected TEM/STEM FEI Titan Themis 300 to examine CNTs at higher magnification after separation from the substrate. Raman spectroscopy, conducted with a HORIBA instrument, was used to assess the quality of CNTs by analyzing their defect density and structural characteristics.

#### 4.2.3 Dope Preparation and Spinning of PAN/CNT Composite Fibers

First, 55 mg PAN powder was dissolved in 220 mL DMF at 90 °C. An equal amount of 55 mg MWCNTs was then dispersed into the polymer solution and sonicated for 24 h using a bath sonicator (Fisher FS30, frequency 43 kHz, power 150 W). The same amounts of PAN and CNTs were chosen in the above processing because of the strong interfacial interactions between the polymer and CNTs that can from in dilute PAN solutions, which is critical for achieving high mechanical reinforcement and performance. Following sonication, vacuum distillation was employed to remove half of the DMF solvent volume under controlled conditions of 90 °C for 5 min. Subsequently, the system was cooled to 60 °C, and nonsolvent DI water (also at 60 °C) was added to the dispersion at a solvent/nonsolvent ratio of 1/2, followed by stirring for 24 h on a hot plate. After equilibrating at 60 °C, the system was cooled to room temperature and filtered through a 0.45 μm nylon membrane (Millipore) under controlled vacuum. The filtered CNT-rich paste was subsequently added to a separate 11 wt % PAN-DMF solution to prepare the final PAN–CNT composite dope. The CNT content in the paste after filtration was approximately 51.75 mg per batch. For a polymer concentration of 11 wt %, the CNT content is 1.5 wt %.^54^ To ensure complete dissolution, the mixture was stirred with an overhead mechanical stirrer at 350 rpm for 1 h.^51^ This process yielded the spinning dope used to fabricate the PAN-CNT composite fibers in this study.

During the wet-spinning process, methanol is continuously pumped into plastic tubing with an inner diameter of 3 mm and maintained at room temperature as the coagulation medium. The PAN–CNT precursor solution is injected into the methanol-filled tubing at a rate of 0.3 mL/min using a syringe pump, delivered through a 20 mL stainless steel syringe fitted with a 22-gauge Kel-F Hub needle (Hamilton Co., catalog number 7750-13). To ensure stable fiber formation, the methanol flow rate is precisely controlled, maintaining a consistent coagulation environment. Upon entering the flowing methanol, the precursor solution immediately forms as-spun fibers, which are collected by take-up rollers at a speed of approximately 5 m/min. Key processing parameters, including methanol flow rate, precursor injection rate, and take-up roller speed, are carefully optimized to achieve consistent fiber morphology, uniform coagulation, and controlled microstructural development.^36,38,54,55^

#### 4.2.4 Characterization of PAN/CNT Composite Fibers

Static tensile tests were conducted using a dynamic mechanical analyzer (DMA) tester with a loading gap of 15 mm (RSA-G2, TA Instruments), and the data from these tests were collected with TRIOS software (TA Instruments). DSC was carried out on different paste film samples using a DSC25 (TA Instruments) with a T-Zero pan and lid. Samples were equilibrated at 25 °C, then ramped to 400 °C at a rate of 10 °C min^–1^ under a nitrogen atmosphere. To determine the fiber weight percentage, thermogravimetric analysis (TGA) was performed using a SDT 650 system (TA Instruments), heating the samples to 1000 °C at 20 °C/min under a nitrogen atmosphere. XRD analysis was conducted using a Rigaku Ultima III X-ray diffractometer. Data conversion and analysis were performed using Jade software.

## Supplementary Material



## Notes

Supporting Information includes detailed synthesis conditions for MWCNTs via catalytic CVD using LDPE feedstock, along with a comprehensive study on catalyst reuse, featuring SEM and TEM analyses of MWCNTs across multiple growth cycles. EDX spectra are provided for mixed CNTs, SWNTs, and the synthesized MWCNTs. The mechanical reinforcement effects of CNTs in PAN composite fibers are evaluated through morphology analysis, tensile testing, and optical microscopy, with additional investigations into the effects of acid treatment and nitrogen flow rate on CNT quality and fiber performance. A comparative analysis between the PAN/CNT fibers developed in this study and those reported by other research groups is also included. Supplementary tables summarize the chemical composition of SS316 catalyst substrates, and the mechanical properties of composite fibers fabricated from various CNT sources (PDF)

## ○

Dr. Marilyn Minus passed away on Tuesday, August 6, 2024, at the age of 46, all her life focused on researching the fabrication and characterization of advanced high-performance polymer nanocomposites.
