# Supplementary material for: Circular Carbon Nanotube Production for Advanced Structural Fiber Applications
Source: ACS Appl Mater Interfaces. 2025 Nov 20;17(48):65848–61. doi: 10.1021/acsami.5c16868 (PMC12679545; doi:10.1021/acsami.5c16868)
Supplement: Supplementary file 1 [file am5c16868_si_001.pdf]

Supporting Information

Circular Carbon Nanotube Production for Advanced Structural Fiber Applications

Xiao Sun[1, &], Di Chang[1, &], Varunkumar Thippanna[2, &], Xiaoli Li[1], Aidin Panahi[1,3], Huidong Dai[4], Hongyi Wang[5], Jianlin Li[1], Yunzheng Yang[1], Arunachalam Ramanathan[2], Wentao Liang[6], Yiannis Levendis[1,], Kenan Song[7,*], Marilyn Minus[1,*†]*

1 Department of Mechanical and Industrial Engineering, Northeastern University, 360 Huntington Avenue, Boston, Massachusetts, 02115, United States
2 Mechanical Engineering, College of Engineering, University of Georgia, 302 E Campus Rd, Athens, 30602, GA, United States
3 Chemical Engineering Department, Worcester Polytechnic Institute, Worcester, Massachusetts, 01609, United States
4 Department of Chemistry and Chemical Biology, Northeastern University, Boston, MA, 02115, United States
5 Kennedy College of Sciences, University of Massachusetts Lowell, 220 Pawtucket Street, Lowell, Massachusetts, 01854, United States
6 Kostas Advanced Nanocharacterization Facility (KANCF), Northeastern University, Burlington, Massachusetts, 01803, USA
7 Mechanical Engineering, College of Engineering, University of Georgia (UGA), 302 E. Campus Rd., Athens, 30602, GA, United States
&: X.S., D.C., and V.T. contributed equally to this work.
*Corresponding author: kenan.song@uga.edu; y.levendis@northeastern.edu

† Dr. Minus passed away on Tuesday, August 6, 2024, at the age of 46, all her life focus on researching the fabrication and characterization of advanced high-performance polymer nanocomposites.

Table of Contents

1. LDPE Sintering-baed MWNT CVD Synthesis Conditions and Yield Calculation. 5
2. Catalyst Reuse Effects during the CNT CVD Synthesis from Plastic Waste...................6
2.1. SEM of MWNTs from Different Catalyst Substrate Cycles 6
2.2. TEM of MWNTs from Different Catalyst Substrate Cycles 8
2.3. TGA of MWNTs from Different Catalyst Substrate Cycles 9
2.4. Raman spectra of CNTs separated from mesh 400 substrates..9
2.5. EDX of CNTs from Different Types (mixed CNTs, SWNTs, and our MWNTs) 10
3. CNT Reinforcement Effects in Polymer Composite Fibers...13
3.1. Composite Fiber Morphology Analysis 13
3.2. Acid Treatment Effects on CNT Quality..14
3.3. Nitrogen Flow Rate During Synthesis Effects on CNT Quality 16
3.4. Dispersion of CNT in PAN matrix 16
3.5. State-of-the-art of the PAN/CNT Composite Fiber Mechanical Properties 18
4. References...21

Table S1. Chemical composition (wt.%) of stainless steel 316. 5
Table S2. Raman peak ratio and MWCNTs purity calculation 10
Table S3. Mechanical Properties of Neat PAN and PAN Composites with Upcycled MWCNTs, Commercial SWCNTs, and Mixed CNTs 18
Table S4. Comparison with other studies utilizing different types of CNTs. 18

Figure S1. Photographs showing (a) the size of the catalyst substrate, (b) the SS316 substrates rolled and placed in a holder, and (c) MWCNTs grown on the catalyst substrate. 5
Figure S2. Yields of CNTs (Y_c, Y_p, and Y_{final}) after each use of SS-316 mesh 400 substrates following treatment. 6
Figure S3. SEM images of MWCNTs grown on catalyst substrates after the second and fourth reuse cycles. Images from left to right correspond to magnifications of 500×, 2.5k×, 15k×, and 30k×. 7
Figure S4. TEM images of MWCNTs separated from (a) the catalyst substrate after the second reuse and (b) the catalyst substrate after the fourth reuse (magnifications: 20K). 8
Figure S5. Thermo-gravimetric analysis of nanomaterials generated on 400 mesh stainless steel substrates after sonication in ethanol, comparing the initial use of the catalyst, after six reuses, and after six reuses followed by acid purification to remove residual 9
Figure S6. Raman spectra of CNTs generated on substrates after different numbers of reuse cycles. 10
Figure S7. EDX spectrum of mixed CNTs. 11
Figure S8. EDX spectrum of SWCNTs. 11
Figure S9. EDX spectrum of MWCNTs. 12
Figure S10. Optical microscope images and corresponding diameter comparisons of the cross-sections of PAN-CNT fibers fabricated using CNTs from (a) initial use, (b) second reuse, and (c) fourth reuse of the catalyst. 13
Figure S11. (a) Modulus and (b) strength of PAN-CNT composite fibers using different CNTs synthesized on the initial use, 2nd reuse, and 4th reuse of the catalyst substrate. (c) Modulus and (d) strength comparison of composite fibers fabricated with CNTs from the 4th reuse catalyst, before and after acid wash treatment. 14
Figure S12. Photo of CNTs before and after acid treatment. 15
Figure S13. SEM images of CNTs grown on SS316 catalyst under a nitrogen flow rate of 2 L/min at (a) 500× and (b) 1.5k× magnifications. (c) Modulus and (d) tensile strength of PAN–CNT composite fibers incorporating CNTs synthesized under different nitrogen flow rates: 0.1 L/min and 2 L/min. 16
Figure S14. FIB TEM of PAN-MWCNT composite fibers 17
Figure S15. Stress–strain curves comparing the elongation at break of PAN + commercial mixed CNTs (12%) and PAN + upcycled MWCNTs (17%). 20

1. LDPE Sintering-baed MWNT CVD Synthesis Conditions and Yield Calculation.

Table S1. Chemical composition (wt.%) of stainless steel 316.

Grade	Iron	C	Si	Mn	P	S	Ni	Cr	Mo
316	balance	0.08	1	2	0.045	0.03	10.0-14.0	16-18	2-3

Figure S1 presents photographs of the SS316 stainless steel wire cloth used as the catalyst substrate for MWCNT growth, alongside details of the preparation process. The SS316 mesh was cut into rectangular pieces measuring 7 cm × 3 cm (**Figure S1a**). For CVD synthesis, the substrates were rolled and secured in a holder to facilitate uniform gas exposure during pyrolysis and CNT growth (**Figure S1b**). After the CVD process, the MWCNT-coated substrates are shown in **Figure S1c**, where the dark surface coating confirms the successful growth of MWCNTs directly on the stainless-steel mesh. Table S1 provides the detailed chemical composition of SS316, highlighting the presence of Fe, Ni, Cr, and Mo, which are critical for catalytic activity in CNT synthesis.

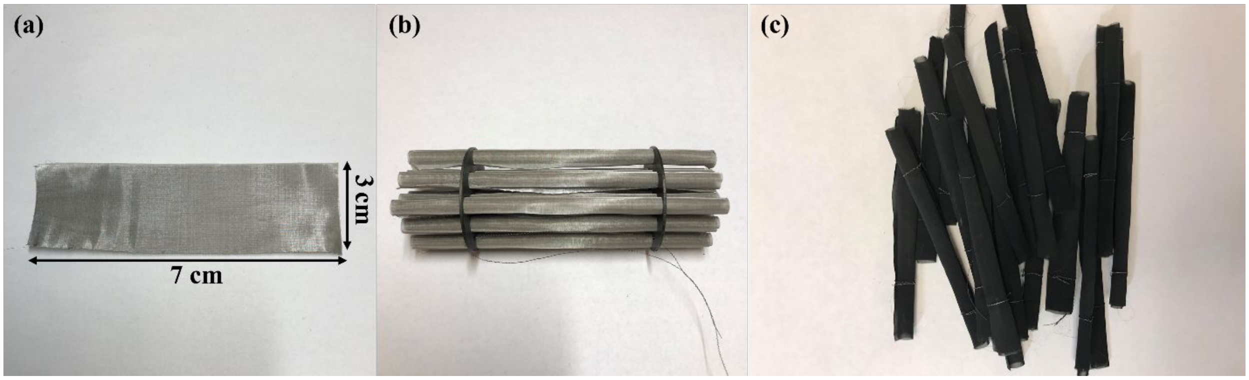

Figure S1. Photographs showing (a) the size of the catalyst substrate, (b) the SS316 substrates rolled and placed in a holder, and (c) MWCNTs grown on the catalyst substrate.

Figure S2 shows the yields of the generated CNTs, expressed on a mass basis, calculated using three different methods according to the formulas provided below.

$$Y_c(\%) = \frac{Mass\ of\ CNT}{Mass\ of\ Catalyst} \times$$

$$Y_p(\%) = \frac{Mass\ of\ CNT}{Mass\ of\ Polymer} \times$$

$$Y_{final}(\%) = \frac{Mass\ of\ CNT\ after\ separation}{Mass\ of\ Polymer} \times$$

Figure S2 illustrates the evolution of CNT yields from the initial use to the sixth reuse. During the first three reuses, Y_c, Y_p, and Y_{final} increased significantly, with Y_p reaching a peak of approximately 50% in the second reuse. Subsequently, Y_c and Y_p gradually declined during the fourth and fifth reuses and stabilized in the sixth reuse. In contrast, Y_{final} decreased in the fourth reuse but rose markedly in the fifth reuse, followed by a stable stage thereafter.

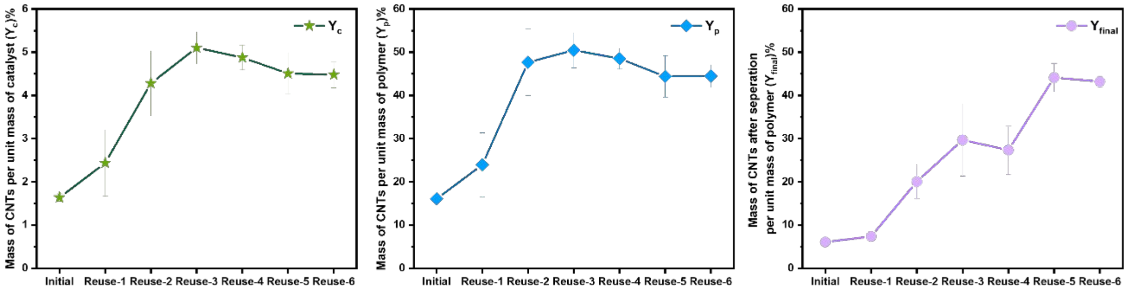

Figure S2. Yields of CNTs (Yc, Yp, and Yfinal) after each use of SS-316 mesh 400 substrates following treatment.

2. Catalyst Reuse Effects during the CNT CVD Synthesis from Plastic Waste

2.1. SEM of MWNTs from Different Catalyst Substrate Cycles

Figure S3 presents SEM images of MWCNTs grown on SS316 catalyst substrates after the second and fourth reuse cycles, highlighting how repeated use of the catalyst affects CNT growth morphology. The images, captured at magnifications ranging from 500× to 30,000×, provide insight into both the catalyst surface evolution and the resulting nanotube quality. In the second reuse cycle (**Figures S3a_1–a_4**), the catalyst surface is already substantially coated with CNTs. At low magnification (S2a_1 and a_2), the images reveal an increasingly textured surface compared to the initial catalyst, indicating significant nanocarbon deposition. At higher magnifications (S2a_3 and a_4), the formation of dense CNT networks becomes evident. These CNTs exhibit relatively uniform diameters, long tube lengths, and less pronounced bundling, suggesting that the catalyst retains good activity and dispersion capability in the early stages of reuse. After the fourth reuse cycle (**Figures S3b_1–b_4**), notable changes in CNT morphology are observed. The catalyst surface shows thicker coverage with carbon deposits, as seen in S2b_1 and b_2. At higher magnifications (**S3b_3** and **b_4**), the MWCNTs display increased entanglement, globular agglomerations, and a broader diameter distribution. There is also evidence of shorter tube lengths and irregular growth patterns, likely due to catalyst surface degradation, deactivation, or site saturation after multiple growth cycles. These effects are consistent with the formation of residual catalyst nanoparticles encapsulated in carbon layers, which can lead to a decline in catalytic uniformity and CNT alignment. The morphological evolution seen here highlights a trade-off between catalyst reusability and CNT quality. While SS316 can be reused multiple times for MWCNT synthesis from plastic-derived carbon sources, repeated cycles tend to compromise nanotube uniformity and structure. This observation suggests that, although catalyst reuse improves process sustainability and cost-effectiveness, periodic regeneration or surface treatment of the catalyst may be necessary to maintain consistent CNT quality over multiple runs in our future research.

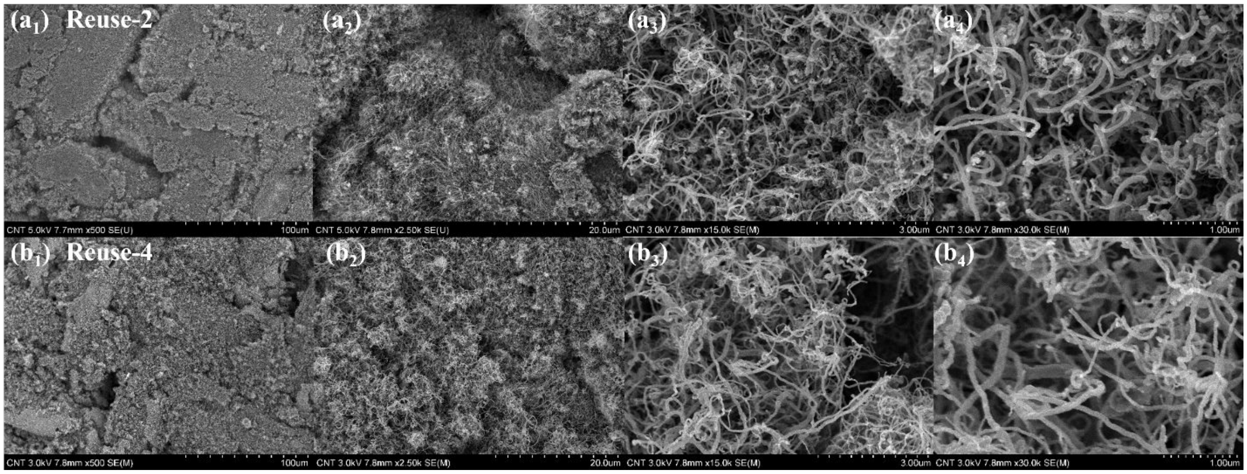

Figure S3. SEM images of MWCNTs grown on catalyst substrates after the (a_1-a_4) second and (b_1-b_4) fourth reuse cycles. Images from left to right correspond to magnifications of 500×, 2.5k×, 15k×, and 30k×.

2.2. TEM of MWNTs from Different Catalyst Substrate Cycles

Figure S4 shows TEM images of our MWCNTs obtained after the second (a) and fourth (b) reuse cycles of the SS316 catalyst at a magnification of 20,000×. After the second reuse, the MWCNTs display relatively uniform tube diameters and well-defined walls with moderate entanglement and visible catalyst particles encapsulated within the tubes. By contrast, the fourth reuse results in more pronounced tube curvature, shorter tube lengths, and increased structural disorder. The accumulation of catalyst residues and irregular carbon deposition suggest a decline in catalyst activity and selectivity with repeated use, leading to less controlled CNT growth.

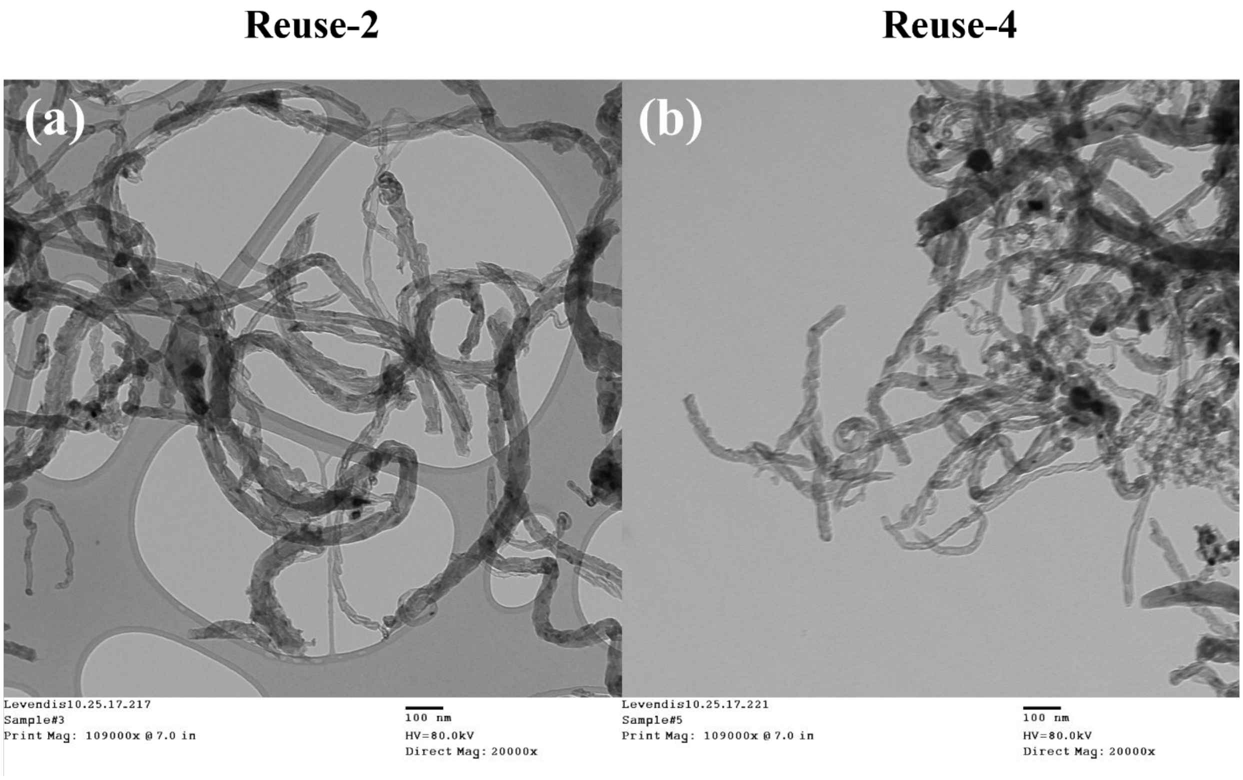

Figure S4. TEM images of MWCNTs separated from (a) the catalyst substrate after the second reuse and (b) the catalyst substrate after the fourth reuse (magnifications: 20K).

2.3. TGA of MWNTs from Different Catalyst Substrate Cycles

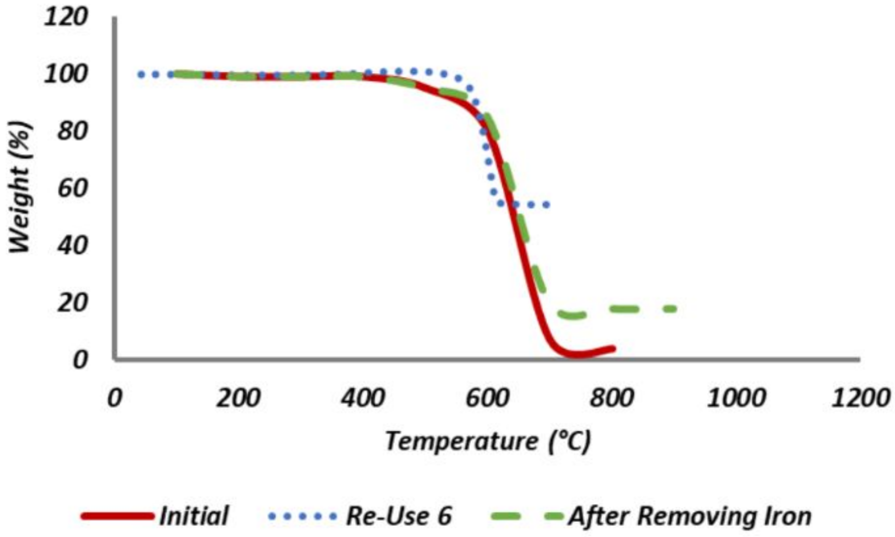

Figure S5. Thermo-gravimetric analysis of nanomaterials generated on 400 mesh stainless steel substrates after sonication in ethanol, comparing the initial use of the catalyst, after six reuses, and after six reuses followed by acid purification to remove residual

2.4. Raman spectra of CNTs separated from mesh 400 substrates

Raman spectroscopy was used to evaluate CNT quality, as shown in **Figure S6**. The spectra displayed three characteristic peaks: D (~1350 cm^{-1}, disordered carbon), G (~1580 cm^{-1}, graphitic ordering), and G′ (~2700 cm^{-1}, long-range order). The I_G/I_D ratio indicates structural ordering and purity, while the $I_{G'}/I_G$ ratio is the most sensitive to MWCNT purity. Purity was further assessed following Dileo et al. using three equations[1]:

$$I_D/I_G = 0.96 - 0.0066X(4)$$

$$I_{G'}/I_G = 0.33 + 0.0045X(5)$$

$$I_{G'}/I_D = 0.31 * \exp(0.021X)(6)$$

Where X is the multi-walled purity value of the CNTs.

Based on the three equations described before, the purity of MWCNTs in the collected nanomaterials was calculated, as summarized in **Table S2.** The highest measured purity was 74%, and good consistency among the three Raman intensity ratios (I_D/I_G, $I_{G'}/I_G$, and $I_{G'}/I_D$) was observed only during the initial use of both meshes. The calculated purity values indicate that the MWCNTs contain certain defects and/or that part of the nanocarbon material is not entirely tubular. They may also suggest the presence of amorphous carbon and residual metal particles. Furthermore, the observed decrease in MWCNT purity with repeated reuse of the 400 mesh substrates aligns well

with the SEM observations, which show increased impurities and more tortuous CNT structures after multiple reuse cycles.

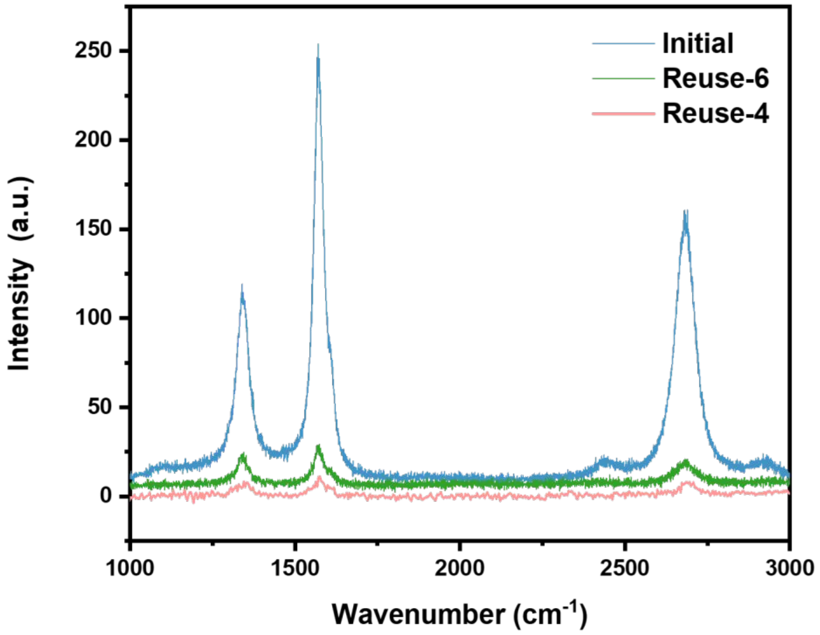

Figure S6. Raman spectra of CNTs generated on substrates after different numbers of reuse cycles.

Table S2. Raman peak ratio and MWCNTs purity calculation

Condition	ID/IG MWNT	Purity (ID/IG)	IG'/IG MWNT	Purity (IG'/IG)	IG'/ID MWNT	Purity (IG'/ID)	Average Purity
Initial	0.44	78%	0.65	70%	1.46	74%	74%
Reuse-4	0.73	35%	0.72	86%	0.99	55%	59%
Reuse-6	0.81	22%	0.64	68%	0.78	44%	45%

2.5. EDX of CNTs from Different Types (mixed CNTs, SWNTs, and our MWNTs)

Figure S7 shows the EDX spectrum of the same region, with strong peaks for carbon and cobalt, along with minor peaks for molybdenum (Mo) and Cr. These results confirm the presence of cobalt-based catalyst residues within the mixed CNTs after synthesis.

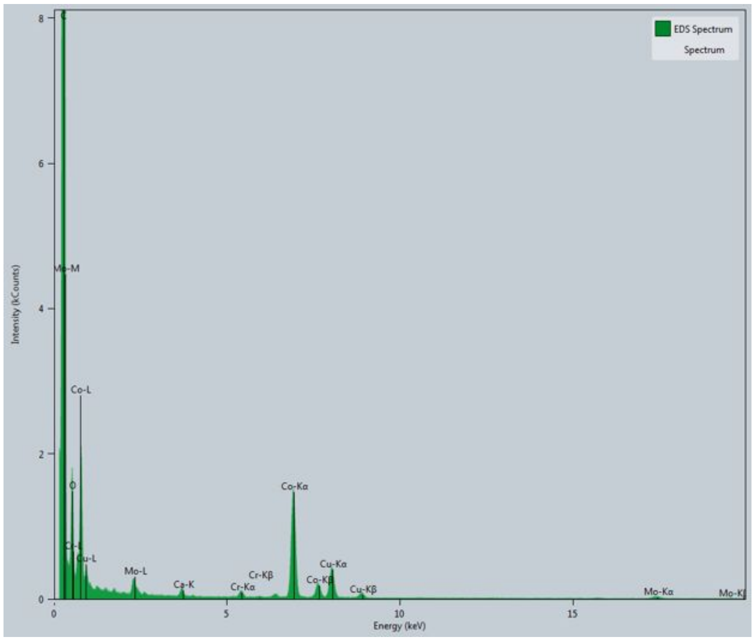

Figure S7. EDX spectrum of mixed CNTs.

The Fe particles is confirmed by the EDX spectrum in **Figure S6**, which shows prominent Fe peaks alongside carbon. Minor copper signals originate from the TEM grid.

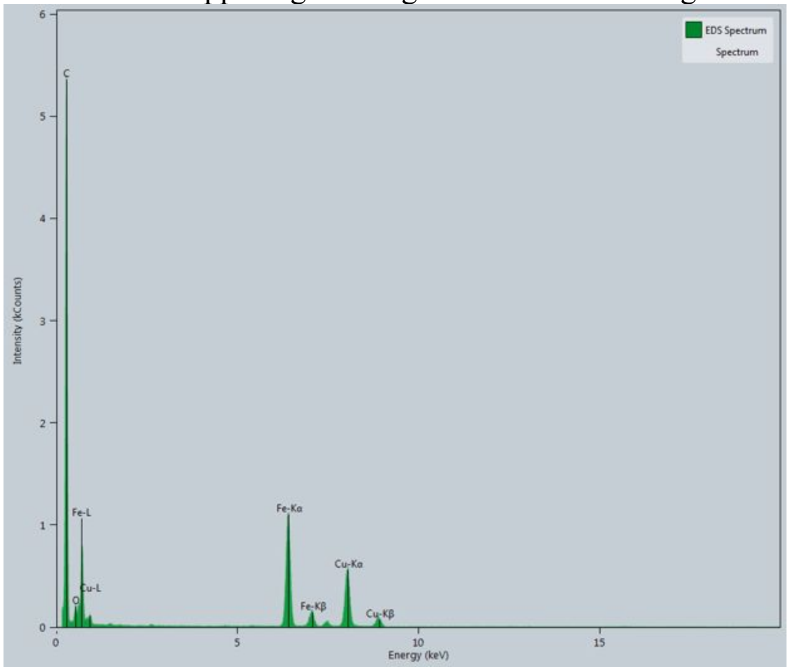

Figure S8. EDX spectrum of SWCNTs.

The EDX map (**Figure S8**) reveals only a few localized signals of iron (Fe, red), indicating minimal metallic residue. This suggests that our CVD synthesis process produced relatively clean MWCNTs with limited catalyst contamination, showing a dominant carbon peak and only weak Fe signals.

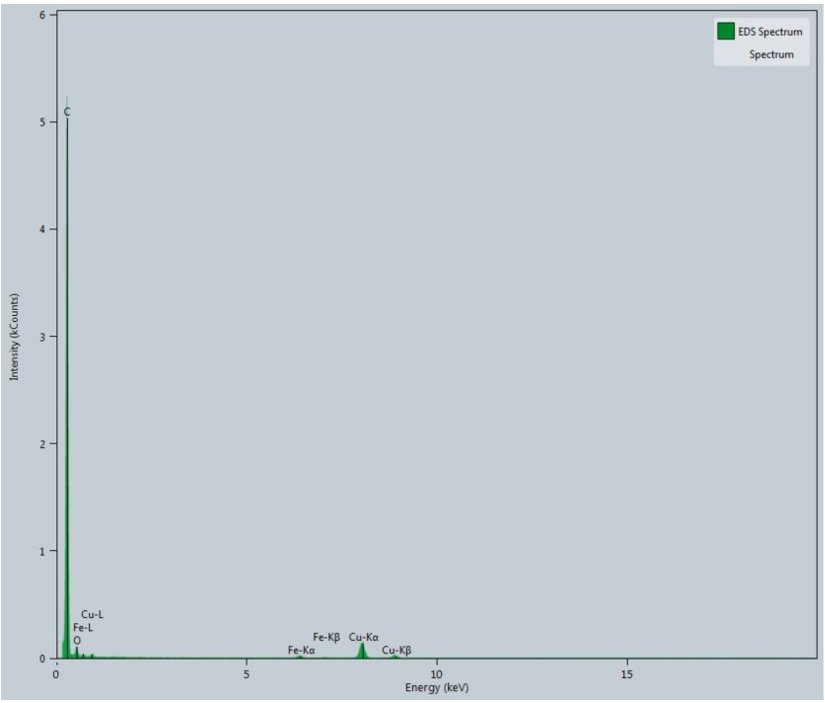

208
209 **Figure S9.** EDX spectrum of MWCNTs.
210

3. CNT Reinforcement Effects in Polymer Composite Fibers

3.1. Composite Fiber Morphology Analysis

Figure S10 presents optical microscope images along with corresponding diameter comparisons of the cross-sections of PAN–CNT fibers fabricated using SS316 catalysts at different reuse stages, including the initial use, second reuse, and fourth reuse.

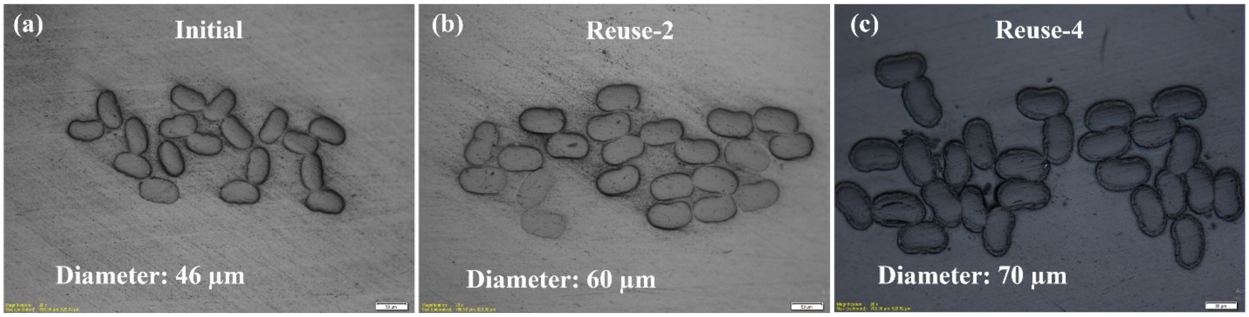

Figure S10. Optical microscope images and corresponding diameter comparisons of the cross-sections of PAN-CNT fibers fabricated using CNTs from (a) initial use, (b) second reuse, and (c) fourth reuse of the catalyst.

3.2. Acid Treatment Effects on CNT Quality

Following the method by Edwards et al., the CNTs were immersed in 38% HCl and sonicated for 1 hour. The CNTs were then filtered using 0.45 µm pore-sized filter paper and rinsed with deionized water until reaching neutral pH.[2] Finally, the CNTs were dried at room temperature.

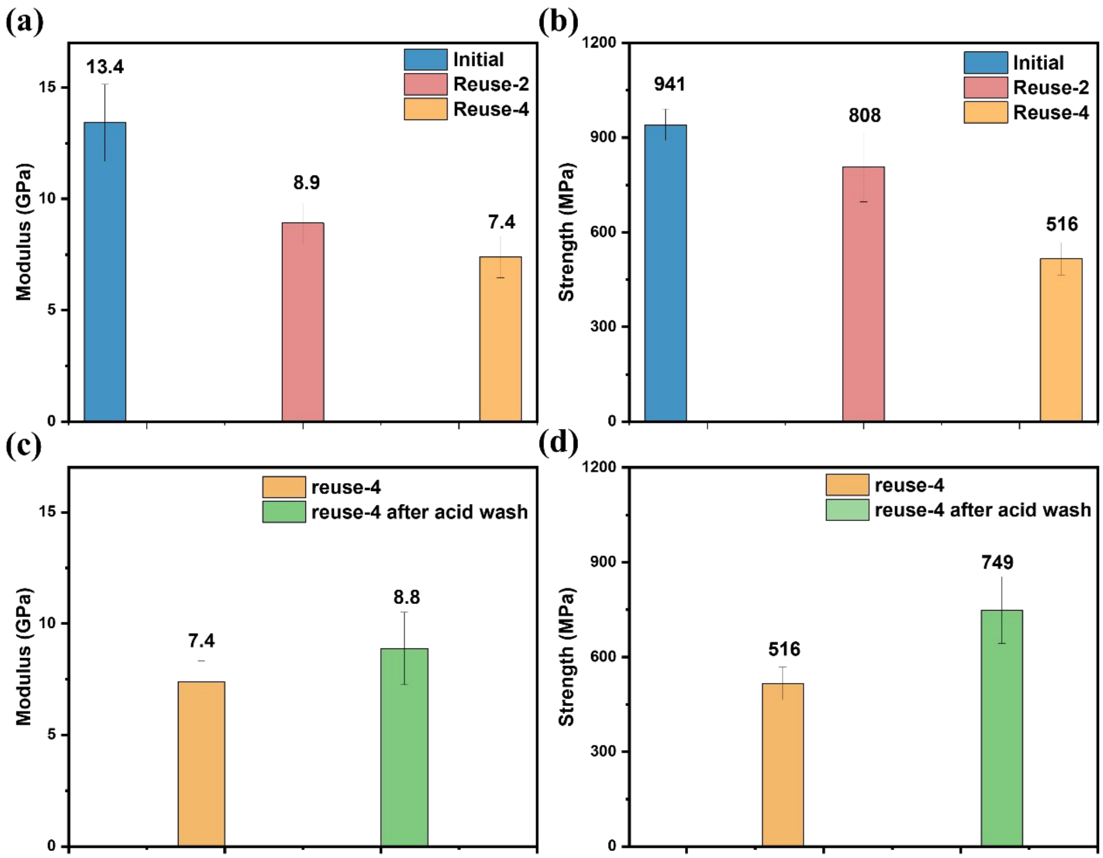

Figure S11. (a) Modulus and (b) strength of PAN-CNT composite fibers using different CNTs synthesized on the initial use, 2nd reuse, and 4th reuse of the catalyst substrate. (c) Modulus and (d) strength comparison of composite fibers fabricated with CNTs from the 4th reuse catalyst, before and after acid wash treatment.

Figure S12 illustrates a visual comparison of CNT dispersions before and after acid treatment. The bottle on the right contains untreated CNTs, while the bottle on the left contains CNTs from the same batch after acid treatment. Both samples were dispersed in isopropyl alcohol, and a magnet was placed between the bottles to demonstrate the removal of magnetic impurities. The photo was taken five seconds after introducing the magnet, clearly showing the effectiveness of the acid purification process.

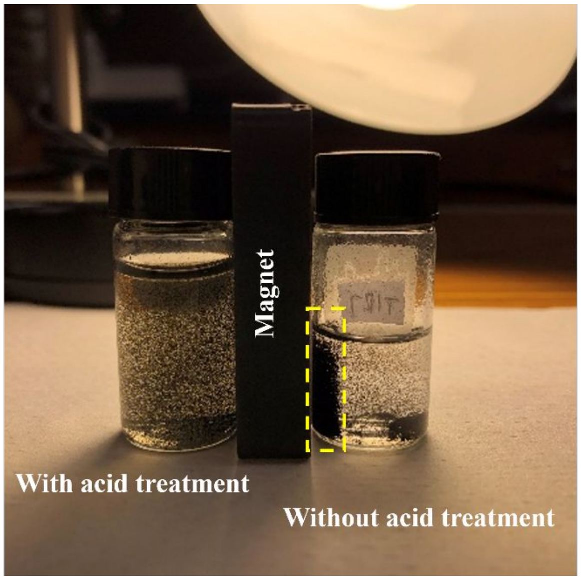

Figure S12. Photo of CNTs before and after acid treatment.

3.3. Nitrogen Flow Rate During Synthesis Effects on CNT Quality

We presented the demonstration of waste plastic-derived MWCNTs directly integrated into PAN composite fibers via industrially mature processes. The resulting fibers not only exhibit enhanced mechanical properties and reduced metal impurity content but also represent a sustainable, scalable solution for high-performance fiber production.

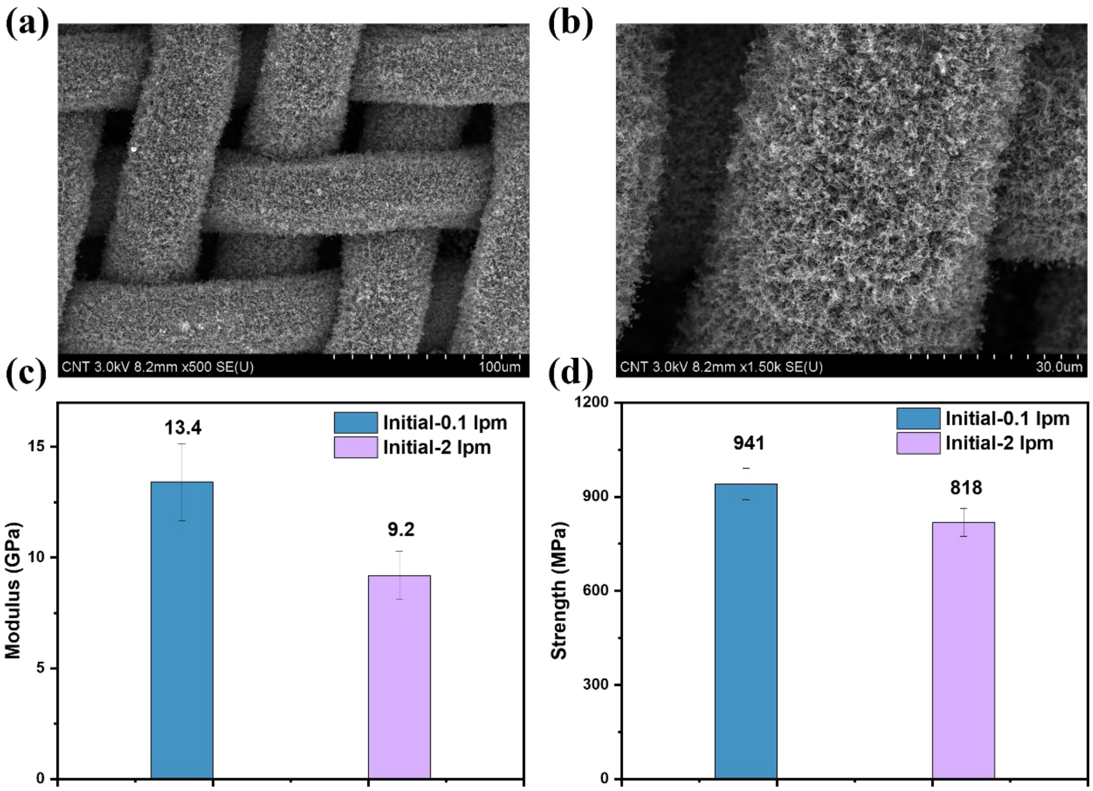

Figure S13. SEM images of CNTs grown on SS316 catalyst under a nitrogen flow rate of 2 L/min at (a) 500× and (b) 1.5k× magnifications. (c) Modulus and (d) tensile strength of PAN–CNT composite fibers incorporating CNTs synthesized under different nitrogen flow rates: 0.1 L/min and 2 L/min.

3.4. Dispersion of CNT in PAN matrix

To verify the effectiveness of the MWCNT dispersion process and ensure that no large aggregates remained, which could lead to defects and non-uniform properties in the composite fibers, the PAN matrix was examined using FIB–TEM. No obvious MWCNT agglomeration was observed, as shown in **Figure S14**. The MWCNTs are well dispersed within the PAN matrix, indicating that the dispersion and mixing procedures were effective. Uniform MWCNT distribution was achieved through a combination of solvent-assisted dispersion and mechanical mixing, as detailed in our previous work.[3] This homogeneous dispersion is crucial for effective load transfer and consistent microstructural properties within the composite fibers, ultimately contributing to improved mechanical performance.

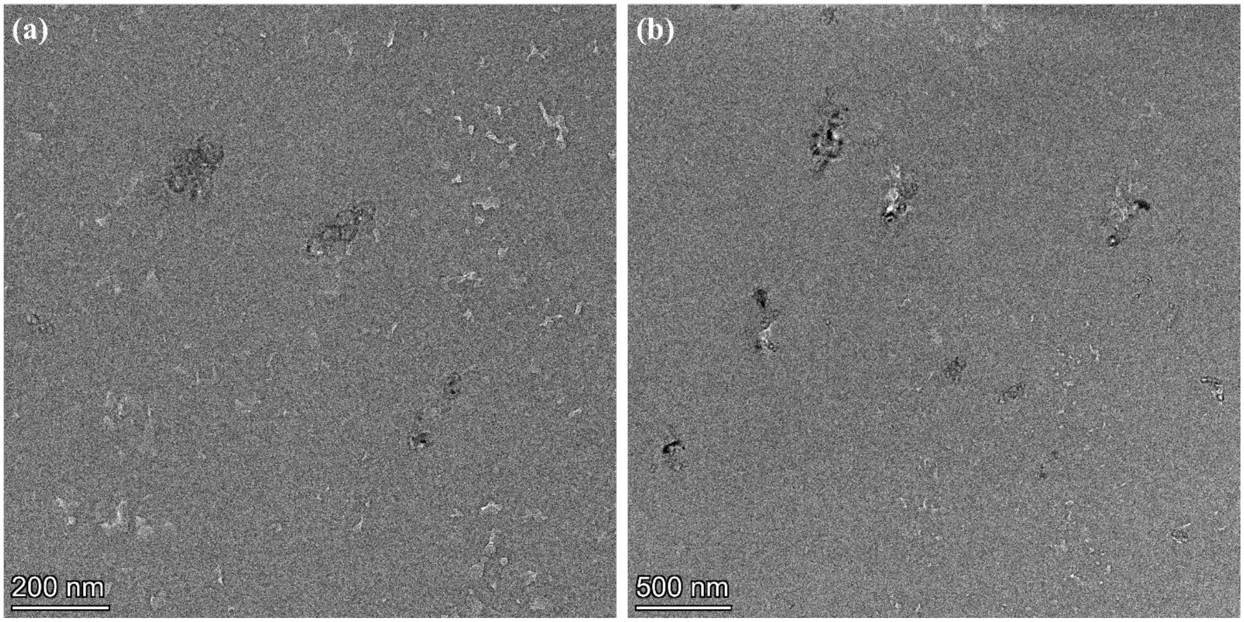

Figure S14. FIB–TEM images of PAN–MWCNT composite fibers: (a) high-magnification image and (b) low-magnification image, showing uniform CNT dispersion within the PAN matrix without noticeable agglomeration.

3.5. State-of-the-art of the PAN/CNT Composite Fiber Mechanical Properties

Table S3. Mechanical Properties of Neat PAN and PAN Composites with Upcycled MWCNTs, Commercial SWCNTs, and Mixed CNTs

CNT types	CNT content (wt.%)	Youngs Modulus (GPa)	Tensile Strength (MPa)
PAN+ upcycle MWCNTs	1.5	13.4	941
PAN+commercial SWCNTs	1.5	13	607
PAN+commercial Mixed CNTs	1.5	12.8	556
Neat PAN	0	8.3	392

Numerous research groups have explored enhancing the performance of PAN fibers by incorporating CNTs, due to their extraordinary mechanical strength, electrical conductivity, and thermal stability.[4-8] Sreekumar et al. fabricated SWCNT/PAN composite fibers containing 0%, 5%, and 10% SWCNTs, reporting that a 10% SWCNT content doubled the tensile modulus at room temperature and increased the storage modulus tenfold at 150 °C, although it resulted in a decrease in tensile strength.[9] In another study, Chae et al. observed that SWCNTs significantly improved the breaking strength, modulus, and failure strain of oxidized PAN fibers.[10] Chae et al. investigated the effects of incorporating 5 wt% of various types of carbon nanotubes including SWNTs, double wall carbon nanotubes (DWNTs), and MWNTs into PAN fibers. SWNTs yielded the greatest improvement in elastic modulus at 75 percent, while MWNTs resulted in the highest increase in tensile strength at 70 percent. The composite fibers showed enhanced PAN orientation and larger crystallite size, with the orientation of the carbon nanotubes significantly higher than that of the PAN matrix. Improvements in low-strain properties were attributed to interactions between PAN and the carbon nanotubes, while enhancements in high-strain properties were partially ascribed to the length of the nanotubes.[10] These improvements were attributed to increased PAN chain orientation and CNT alignment, which are closely related to the surface area and morphology of the CNTs. Although these findings are significant for enhancing composite fiber performance, the issue of catalyst residue in CNTs has not been thoroughly investigated. In this work, the highest tensile strength was achieved, primarily due to the substantially reduced catalyst residue.

Table S4. Comparison with other studies utilizing different types of CNTs.

CNT types	CNT content (wt.%)	Youngs Modulus (GPa)	Tensile Strength (MPa)	Reference
MWCNTs	1.5	13.4	941	This work
SWCNTs	1.5	13	607	
Mixed CNTs (SWCNTs-DWCNTs)	1.5	12.8	556	

MWCNTs	0.5	10.5	822	5
MWCNTs	1	11.4	906	5
SWCNTs	5	13.6	335	10
DWCNTs	5	9.7	316	10
MWCNTs	5	10.8	412	10
SWCNTs	5	14.2	360	9
SWCNTs	10	16.2	330	9
MWCNT	1	12.1	570	11
SWCNT	1	11.5	540	11

Table S4 presents a comparison of the mechanical performance of PAN–CNT composite fibers from this study with previously reported systems using different CNT types and loadings. Notably, the waste-derived MWCNTs used in this work (1.5 wt.%) yield a Young's modulus of 13.4 GPa and a tensile strength of 941 MPa, outperforming both commercial SWCNT- and MWCNT-reinforced fibers at comparable or even higher CNT contents. For instance, other studies using 5–10 wt.% SWCNTs or DWCNTs achieved similar or slightly higher modulus values (up to 16.2 GPa) but with significantly lower tensile strengths (330–360 MPa), highlighting a typical trade-off between stiffness and strength due to CNT aggregation and poor load transfer at higher filler loadings. By applying composite mechanics models such as the rule of mixtures and Halpin-Tsai equations, the superior performance of this study suggests that the intrinsic properties of the waste-derived MWCNTs are closer to their theoretical potential. The higher reinforcement efficiency is attributed to improved interfacial bonding from cleaner CNT surfaces with fewer catalyst residues, superior dispersion in the PAN matrix, and enhanced crystallinity of the composite. While some previous works achieved high stiffness with SWCNTs at higher loadings, their lower strength likely results from interfacial defects and increased CNT entanglement, which act as stress concentrators. In contrast, the current work demonstrates that MWCNTs synthesized from plastic waste can achieve both high modulus and strength at relatively low CNT content (1.5 wt.%), underscoring the dual benefits of sustainable CNT production and efficient reinforcement for advanced fiber applications.

In **Figure S15**, the PAN + commercial mixed CNTs sample shows an elongation at break of approximately **12%**, indicating moderate ductility. In comparison, the PAN + upcycled MWCNTs sample reaches an elongation at break of around **17%**, reflecting a noticeably higher capacity for deformation before failure. This improvement suggests that the upcycled MWCNTs contribute more effectively to enhancing the fiber's extensibility, likely due to better dispersion or interfacial interactions with the PAN matrix, resulting in improved load transfer and structural flexibility. The PAN + upcycled MWCNTs sample exhibits not only a higher elongation at break but also significantly greater toughness compared to the PAN + commercial mixed CNTs sample. This is attributed to its combination of higher tensile strength and larger strain at failure, resulting in a larger area under the stress–strain curve. Quantitatively, the estimated toughness of the PAN + upcycled MWCNTs fiber is approximately twice that of the PAN + commercial mixed CNTs fiber, indicating superior energy absorption capability and improved overall mechanical performance.

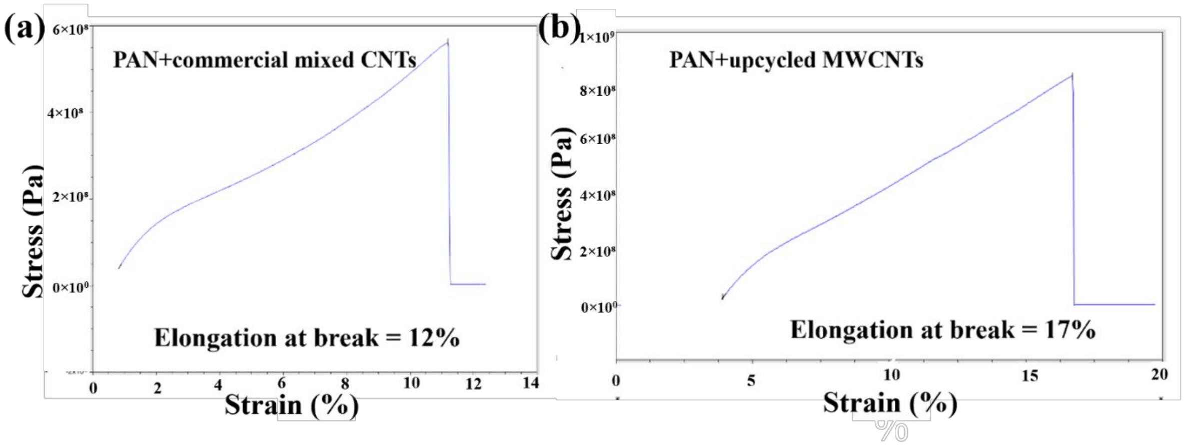

Figure S15. Stress–strain curves comparing the elongation at break of (a) PAN + commercial mixed CNTs (12%) and (b) PAN + upcycled MWCNTs (17%).

4. References

(1) DiLeo, R. A.; Landi, B. J.; Raffaelle, R. P. Purity assessment of multiwalled carbon nanotubes by Raman spectroscopy. *Journal of applied physics* **2007**, *101* (6).

(2) Edwards, E.; Antunes, E.; Botelho, E. C.; Baldan, M.; Corat, E. Evaluation of residual iron in carbon nanotubes purified by acid treatments. *Applied Surface Science* **2011**, *258* (2), 641-648.

(3) Sun, X.; Li, X.; Thippanna, V.; Doyle, C.; Mu, Y.; Barrett, T.; Chambers, L. B.; Yu, C.; Levendis, Y.; Song, K. Carbon Nanoparticle Effects on PAN Crystallization for Higher-Performance Composite Fibers. *ACS Polymers Au* **2025**.

(4) Xu, W.; Ravichandran, D.; Jambhulkar, S.; Zhu, Y.; Song, K. Hierarchically structured composite fibers for real nanoscale manipulation of carbon nanotubes. *Advanced Functional Materials* **2021**, *31* (14), 2009311.

(5) Zhang, J.; Zhang, Y.; Zhang, D.; Zhao, J. Dry-jet wet-spun PAN/MWCNT composite fibers with homogeneous structure and circular cross-section. *Journal of Applied Polymer Science* **2012**, *125* (S1), E58-E66.

(6) Newcomb, B. A.; Chae, H. G.; Gulgunje, P. V.; Gupta, K.; Liu, Y.; Tsentalovich, D. E.; Pasquali, M.; Kumar, S. Stress transfer in polyacrylonitrile/carbon nanotube composite fibers. *Polymer* **2014**, *55* (11), 2734-2743.

(7) Jain, R.; Minus, M. L.; Chae, H. G.; Kumar, S. Processing, structure, and properties of PAN/MWNT composite fibers. *Macromolecular Materials and Engineering* **2010**, *295* (8), 742-749.

(8) Mirbaha, H.; Scardi, P.; D'Incau, M.; Arbab, S.; Nourpanah, P.; Pugno, N. M. Supramolecular structure and mechanical properties of wet-spun polyacrylonitrile/carbon nanotube composite fibers influenced by stretching forces. *Frontiers in Materials* **2020**, *7*, 226.

(9) Sreekumar, T. V.; Liu, T.; Min, B. G.; Guo, H.; Kumar, S.; Hauge, R. H.; Smalley, R. E. Polyacrylonitrile single-walled carbon nanotube composite fibers. *Advanced materials* **2004**, *16* (1), 58-61.

(10) Chae, H. G.; Sreekumar, T.; Uchida, T.; Kumar, S. A comparison of reinforcement efficiency of various types of carbon nanotubes in polyacrylonitrile fiber. *Polymer* **2005**, *46* (24), 10925-10935.

(11) Mikolajczyk, T.; Szparaga, G.; Bogun, M.; Fraczek-Szczypta, A.; Blazewicz, S. Effect of spinning conditions on the mechanical properties of polyacrylonitrile fibers modified with carbon nanotubes. *Journal of applied polymer science* **2010**, *115* (6), 3628-3635.
